# Supplementary figures and images for: Mechanical stimulation promotes enthesis injury repair by mobilizing Prrx1+ cells via ciliary TGF-β signaling
Source: eLife. 2022 Apr 27;11:e73614. doi: 10.7554/eLife.73614 (PMC9094755; doi:10.7554/eLife.73614)

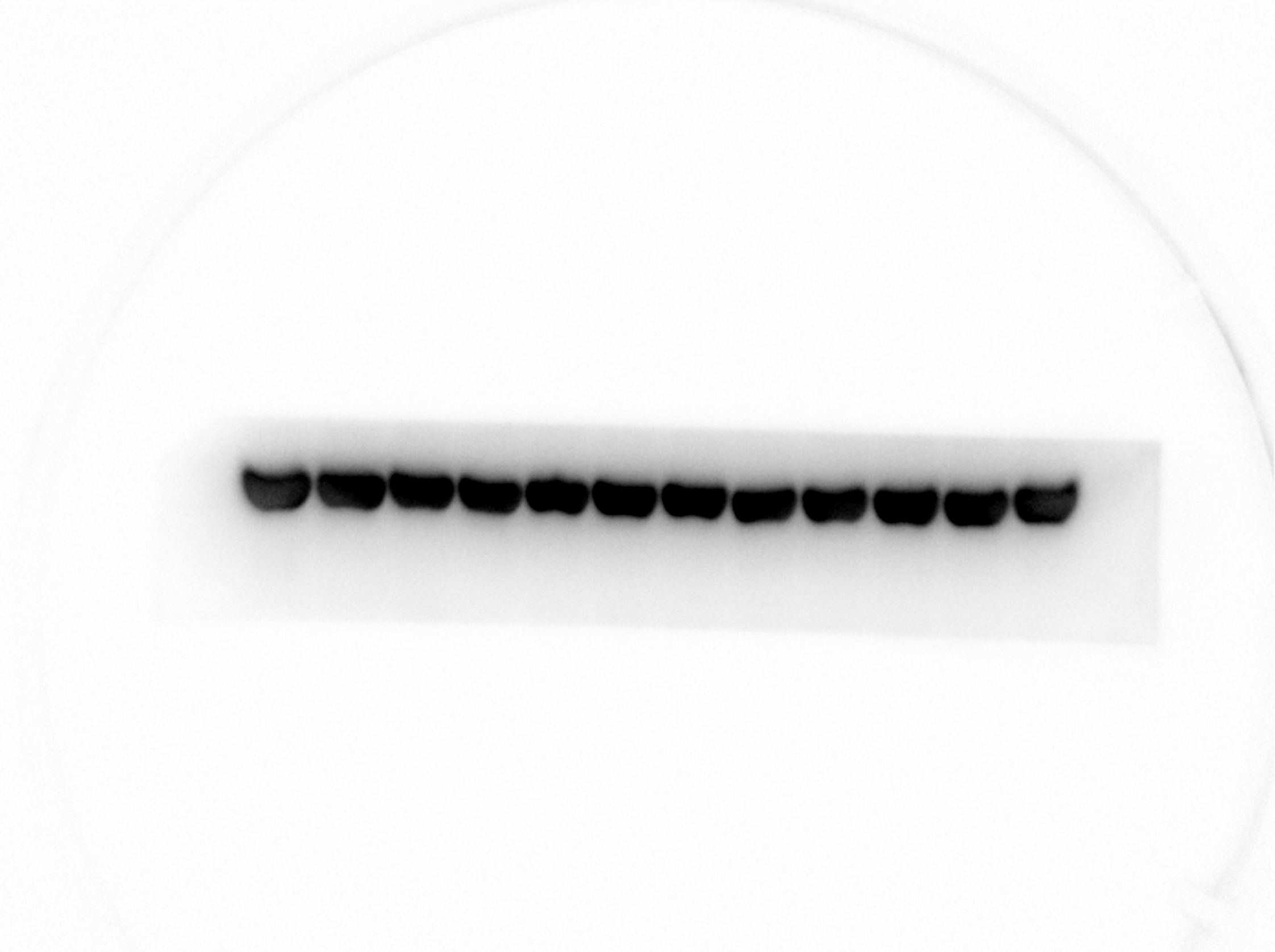

Supplement: Figure 6—figure supplement 1—source data 2. [file elife-73614-fig6-figsupp1-data2.zip › Figure 6S gel/GAPDH 1 Gray.tif]

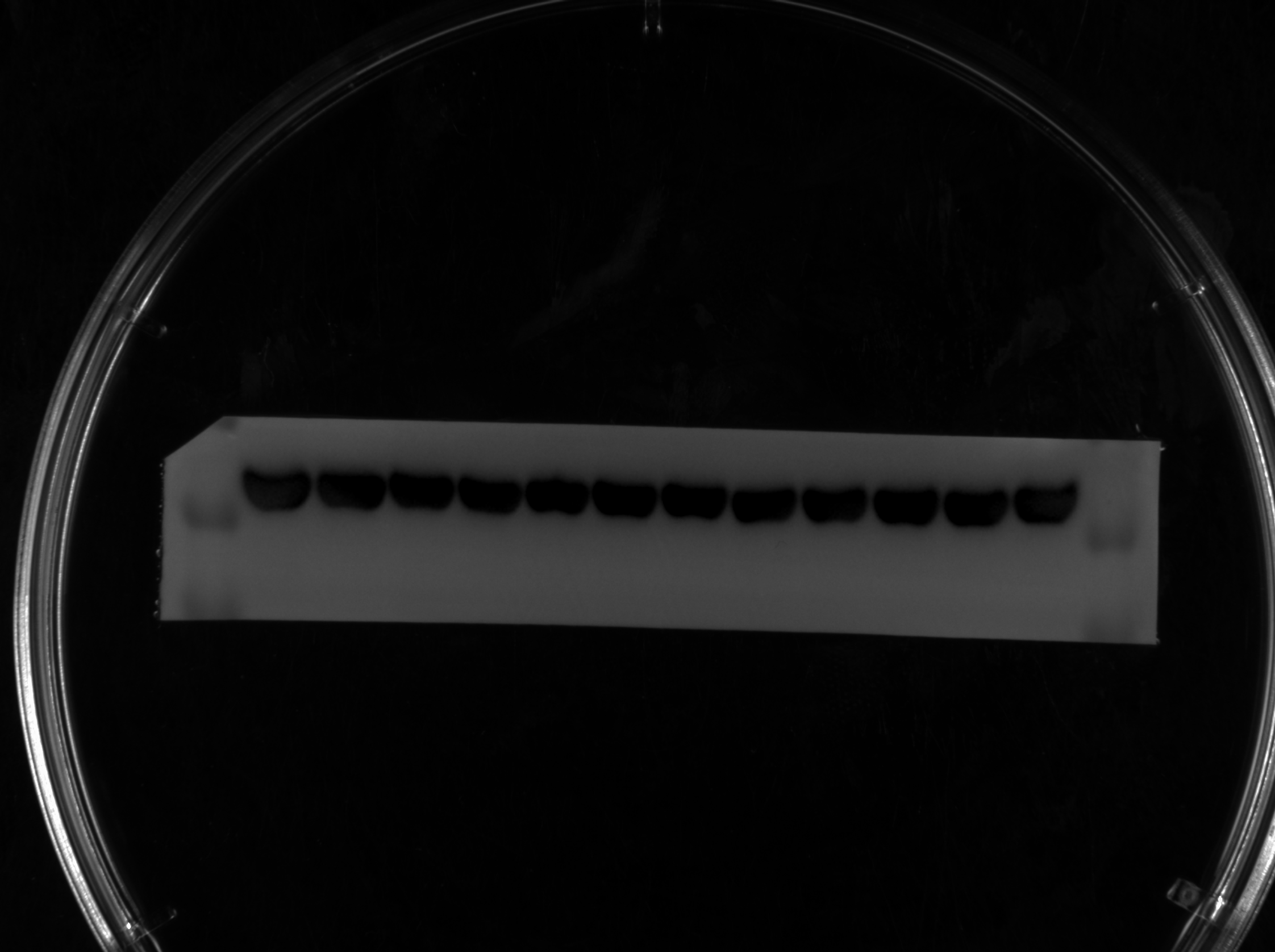

Supplement: Figure 6—figure supplement 1—source data 2. [file elife-73614-fig6-figsupp1-data2.zip › Figure 6S gel/GAPDH 1.tif]

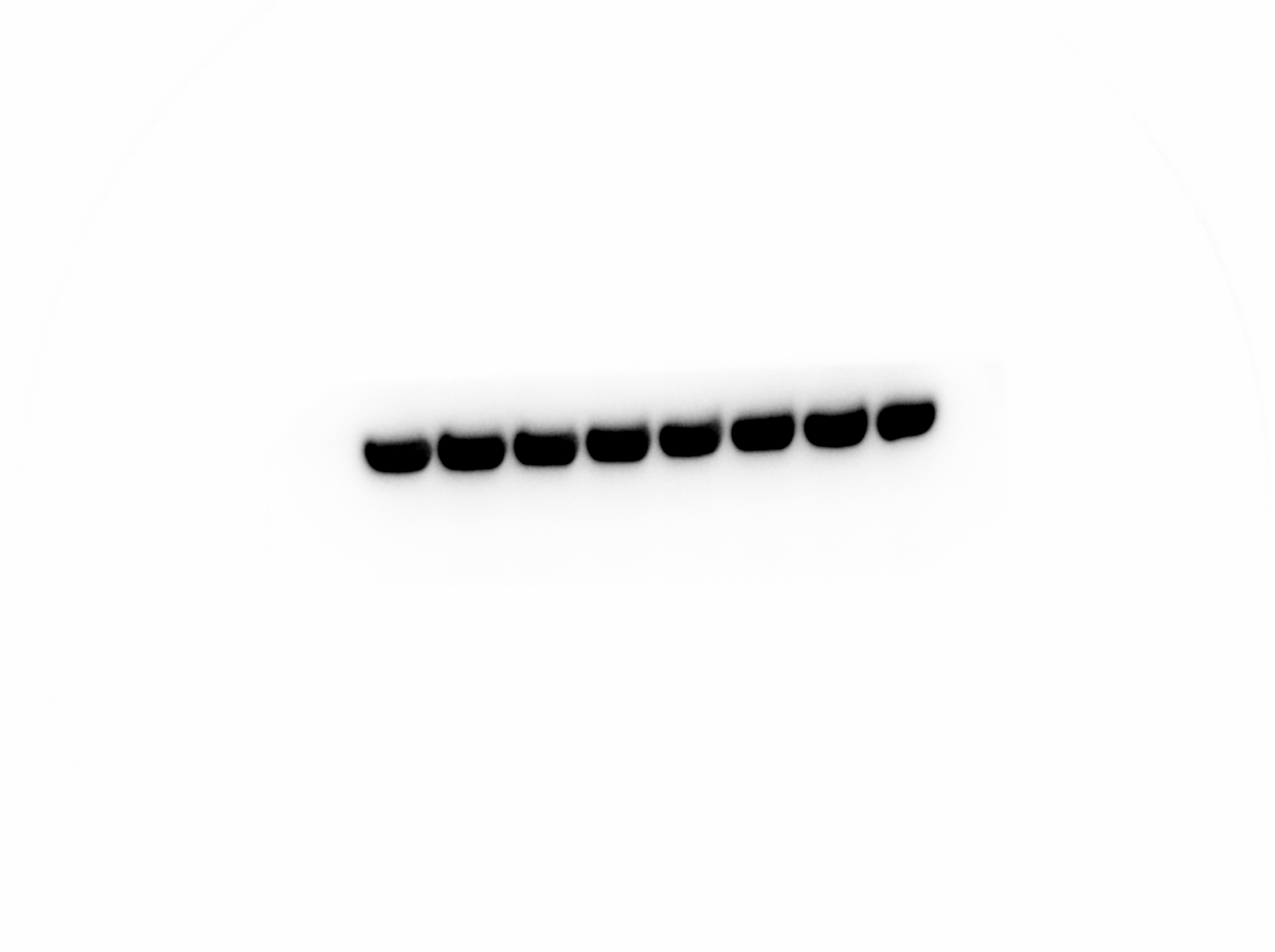

Supplement: Figure 6—figure supplement 1—source data 2. [file elife-73614-fig6-figsupp1-data2.zip › Figure 6S gel/GAPDH 2 Gray.tif]

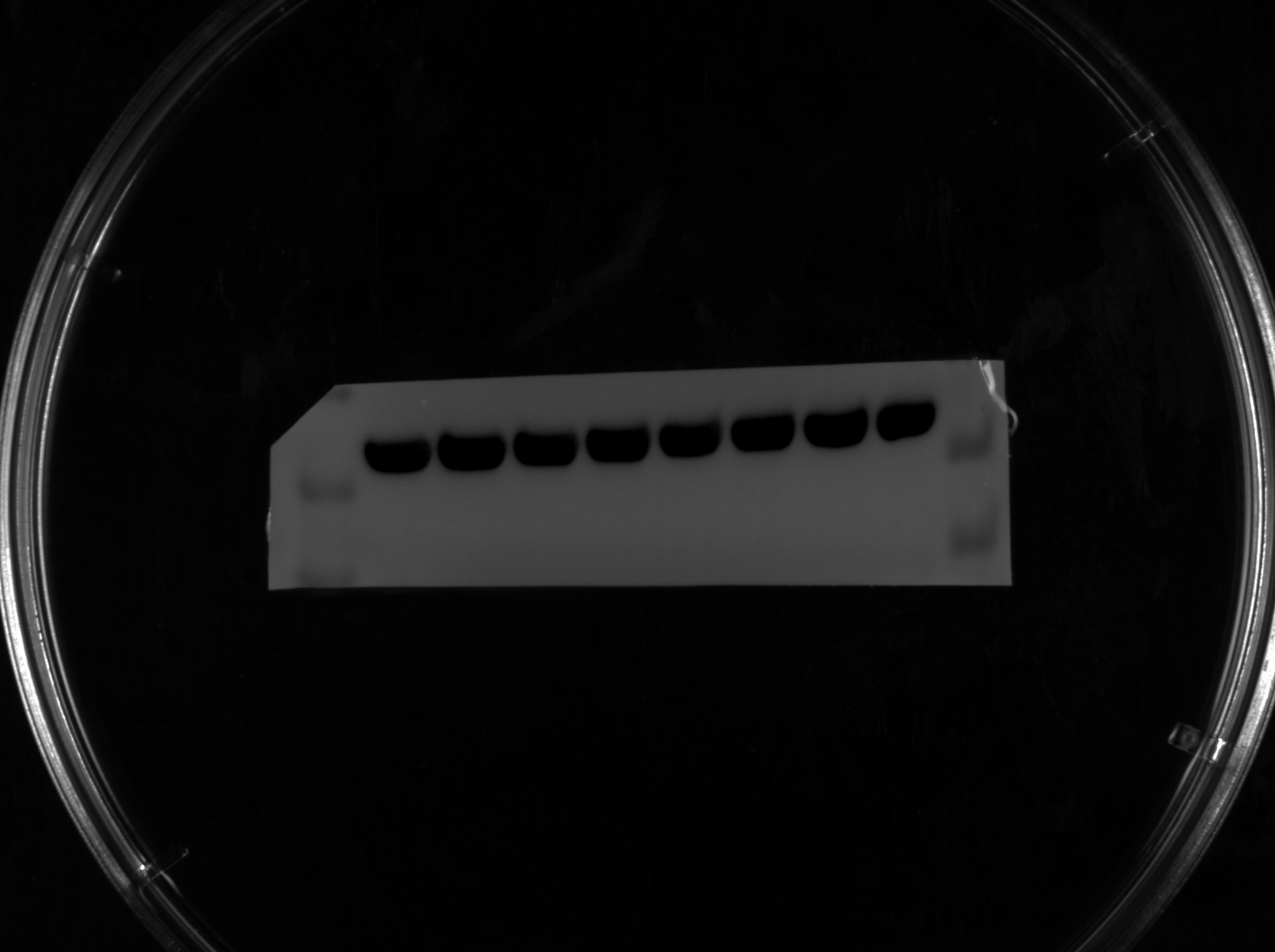

Supplement: Figure 6—figure supplement 1—source data 2. [file elife-73614-fig6-figsupp1-data2.zip › Figure 6S gel/GAPDH 2.tif]

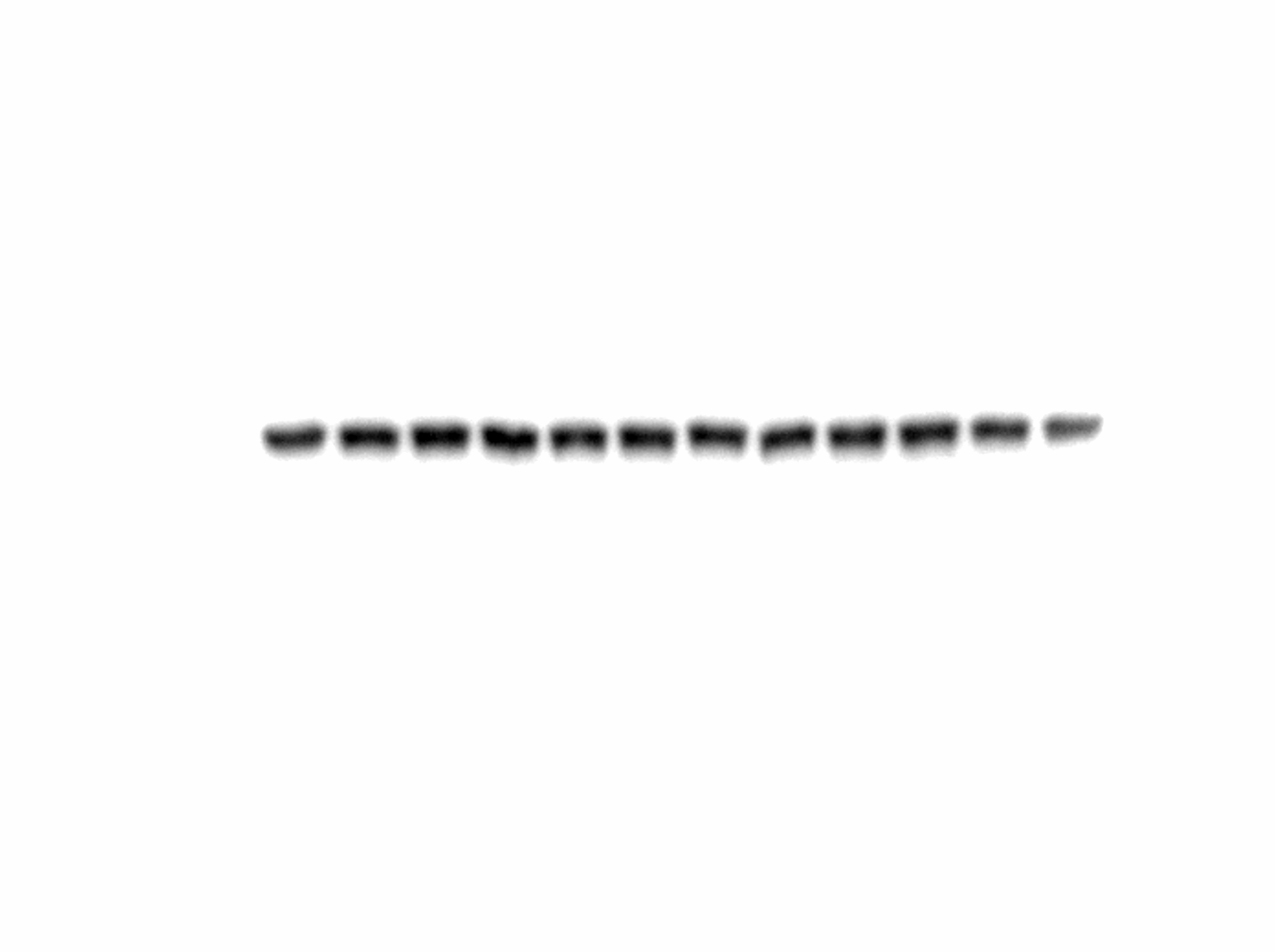

Supplement: Figure 6—figure supplement 1—source data 2. [file elife-73614-fig6-figsupp1-data2.zip › Figure 6S gel/Smad 1 Gray.tif]

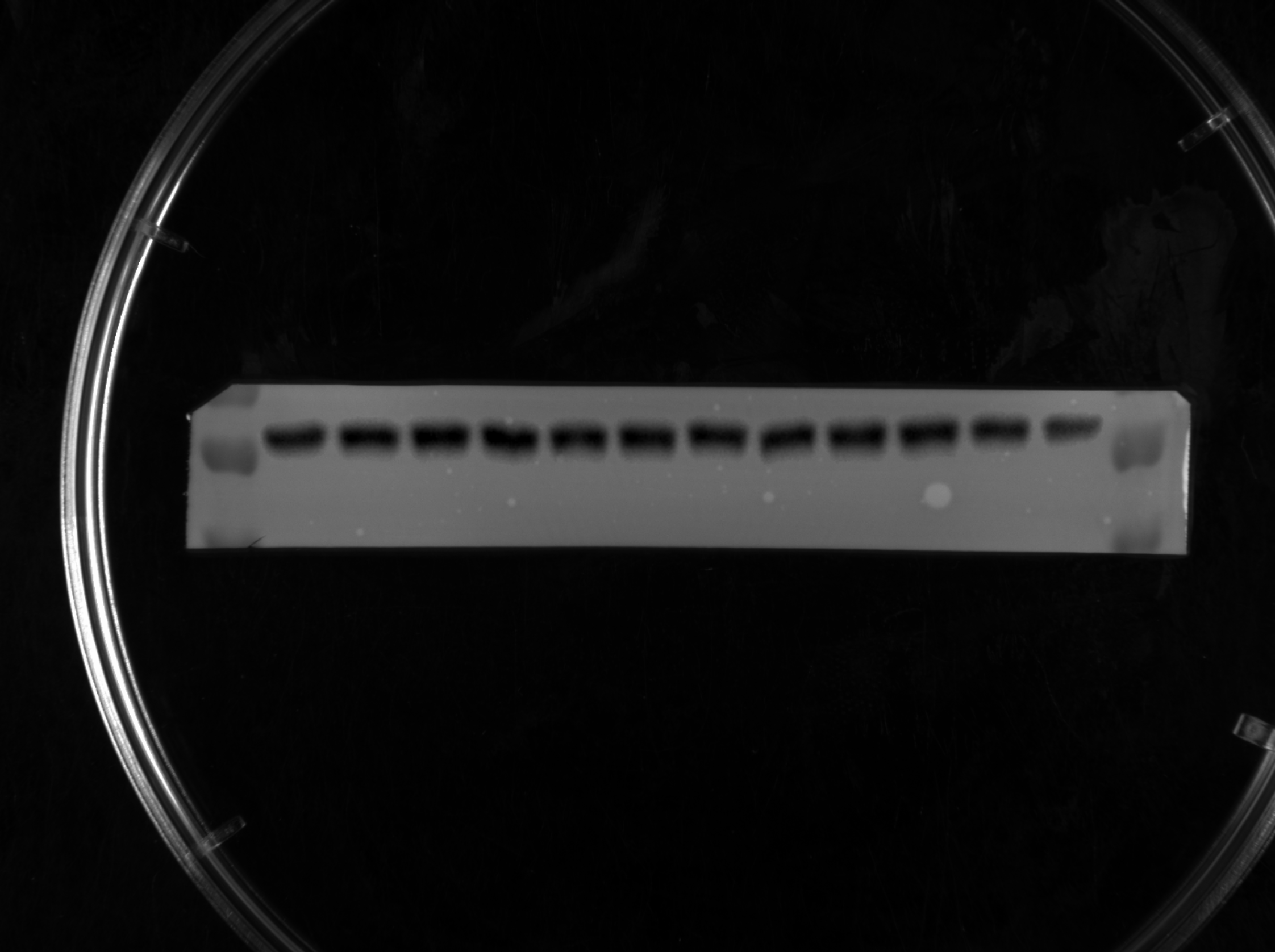

Supplement: Figure 6—figure supplement 1—source data 2. [file elife-73614-fig6-figsupp1-data2.zip › Figure 6S gel/Smad 1.tif]

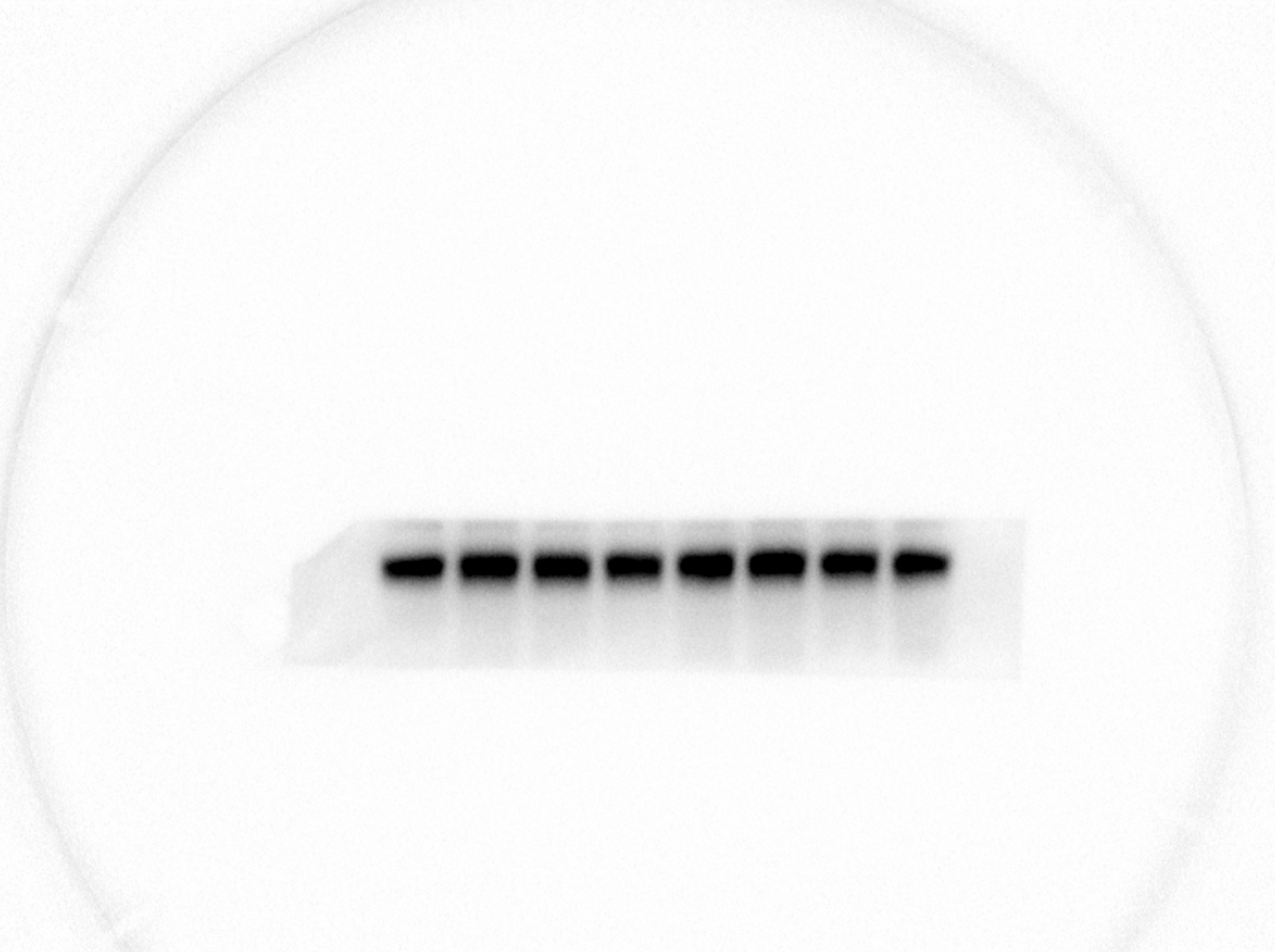

Supplement: Figure 6—figure supplement 1—source data 2. [file elife-73614-fig6-figsupp1-data2.zip › Figure 6S gel/Smad 2 Gray.tif]

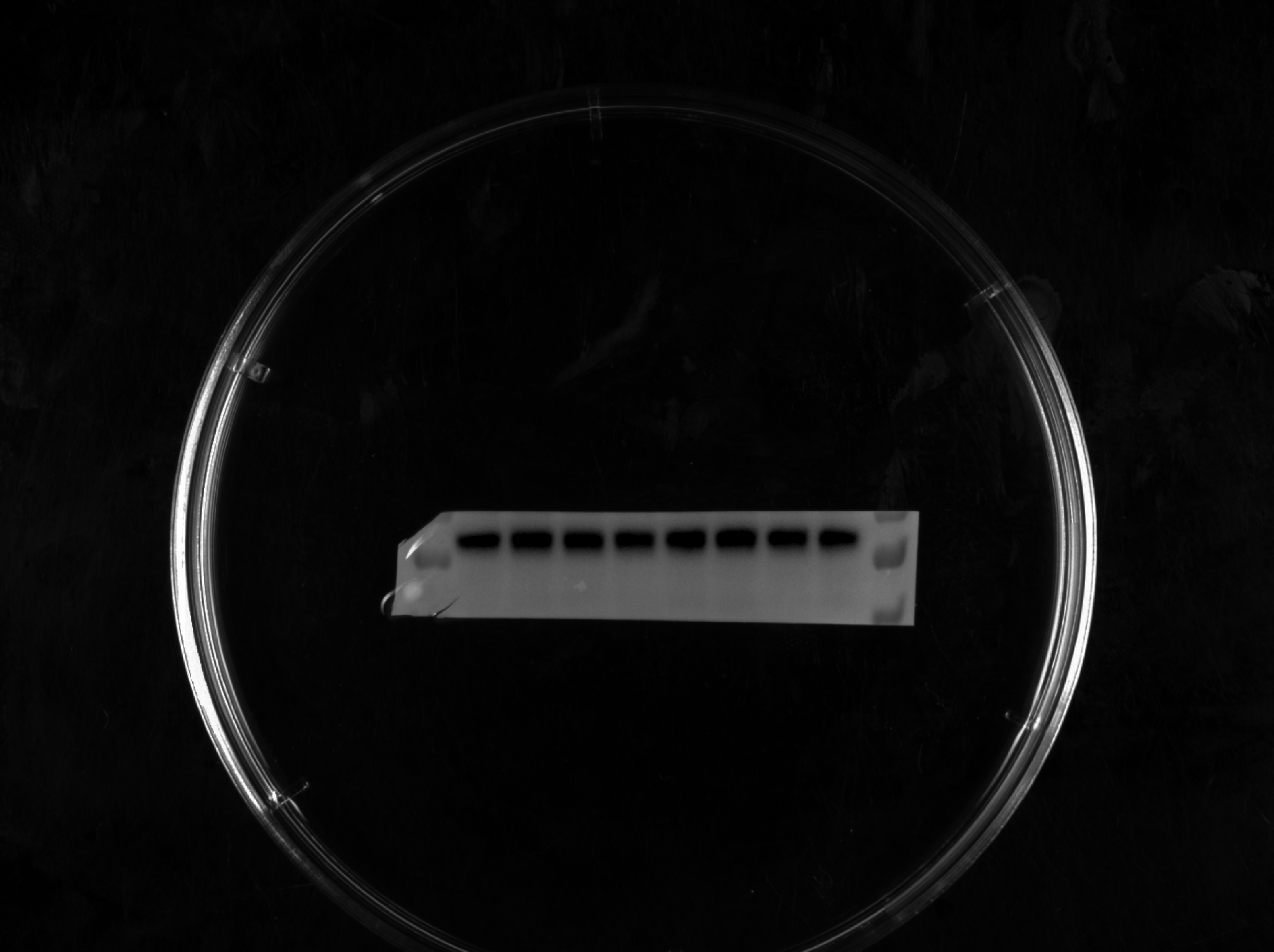

Supplement: Figure 6—figure supplement 1—source data 2. [file elife-73614-fig6-figsupp1-data2.zip › Figure 6S gel/Smad 2.tif]

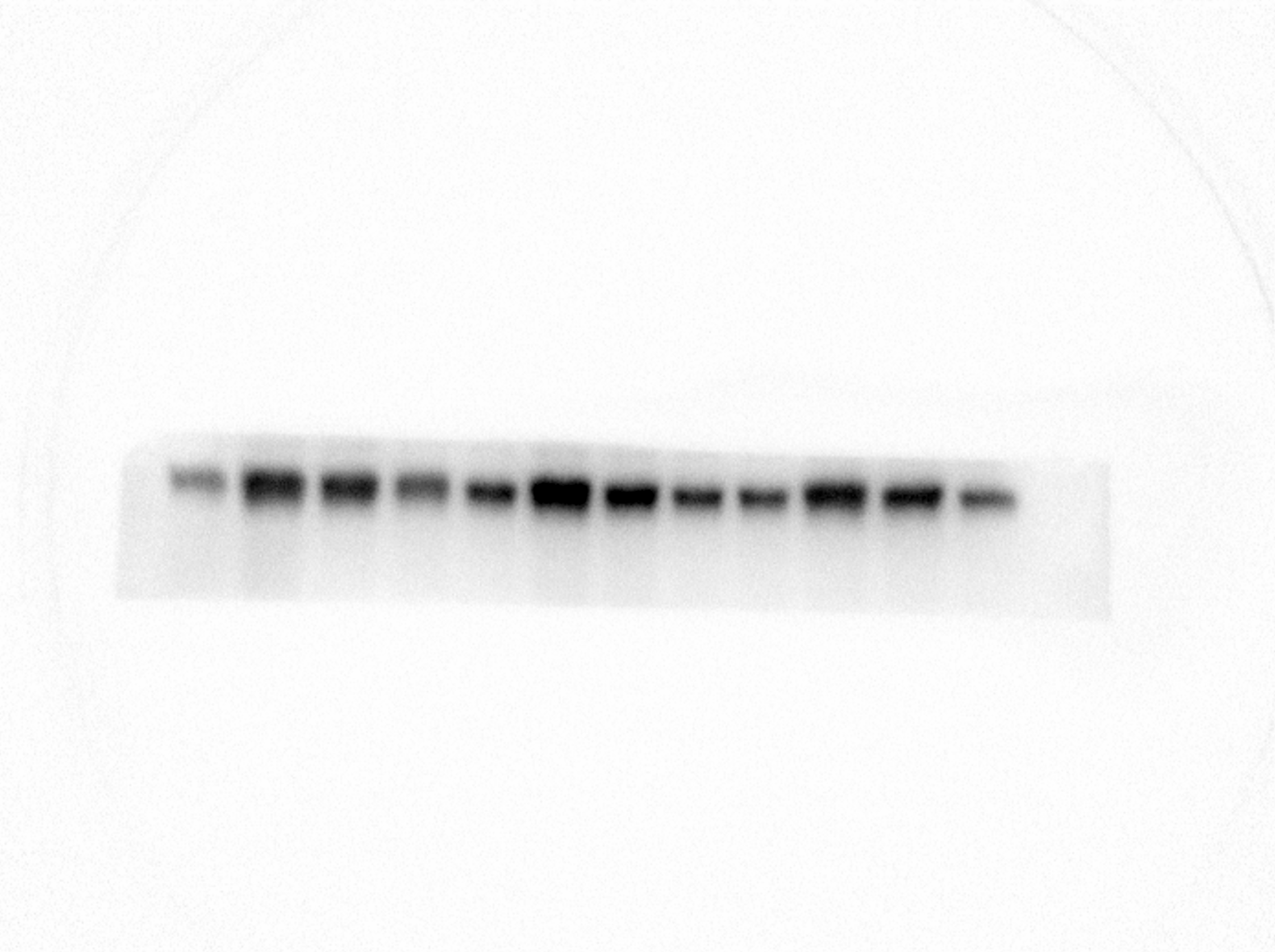

Supplement: Figure 6—figure supplement 1—source data 2. [file elife-73614-fig6-figsupp1-data2.zip › Figure 6S gel/pSmad 1 Gray.tif]

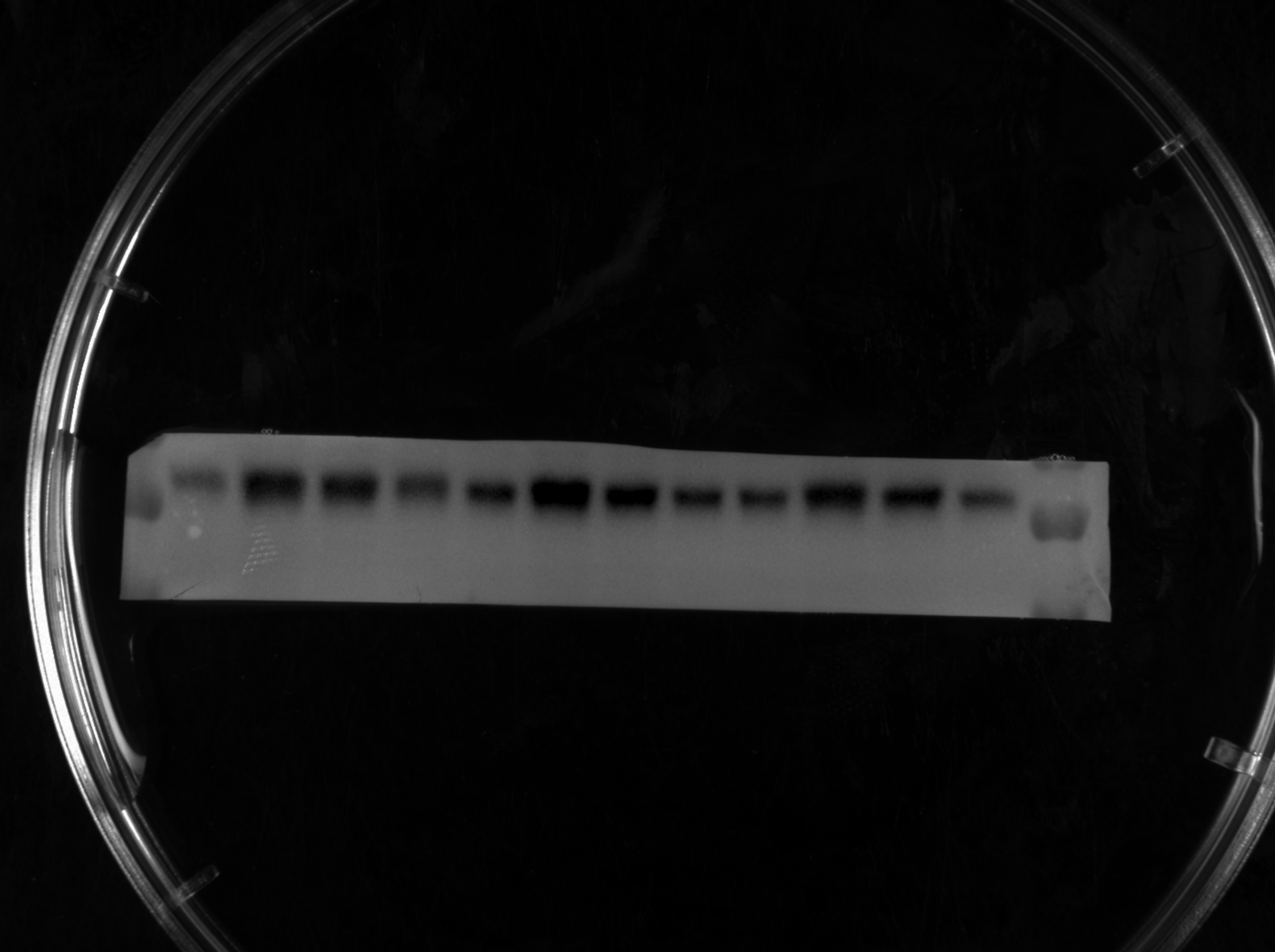

Supplement: Figure 6—figure supplement 1—source data 2. [file elife-73614-fig6-figsupp1-data2.zip › Figure 6S gel/pSmad 1.tif]

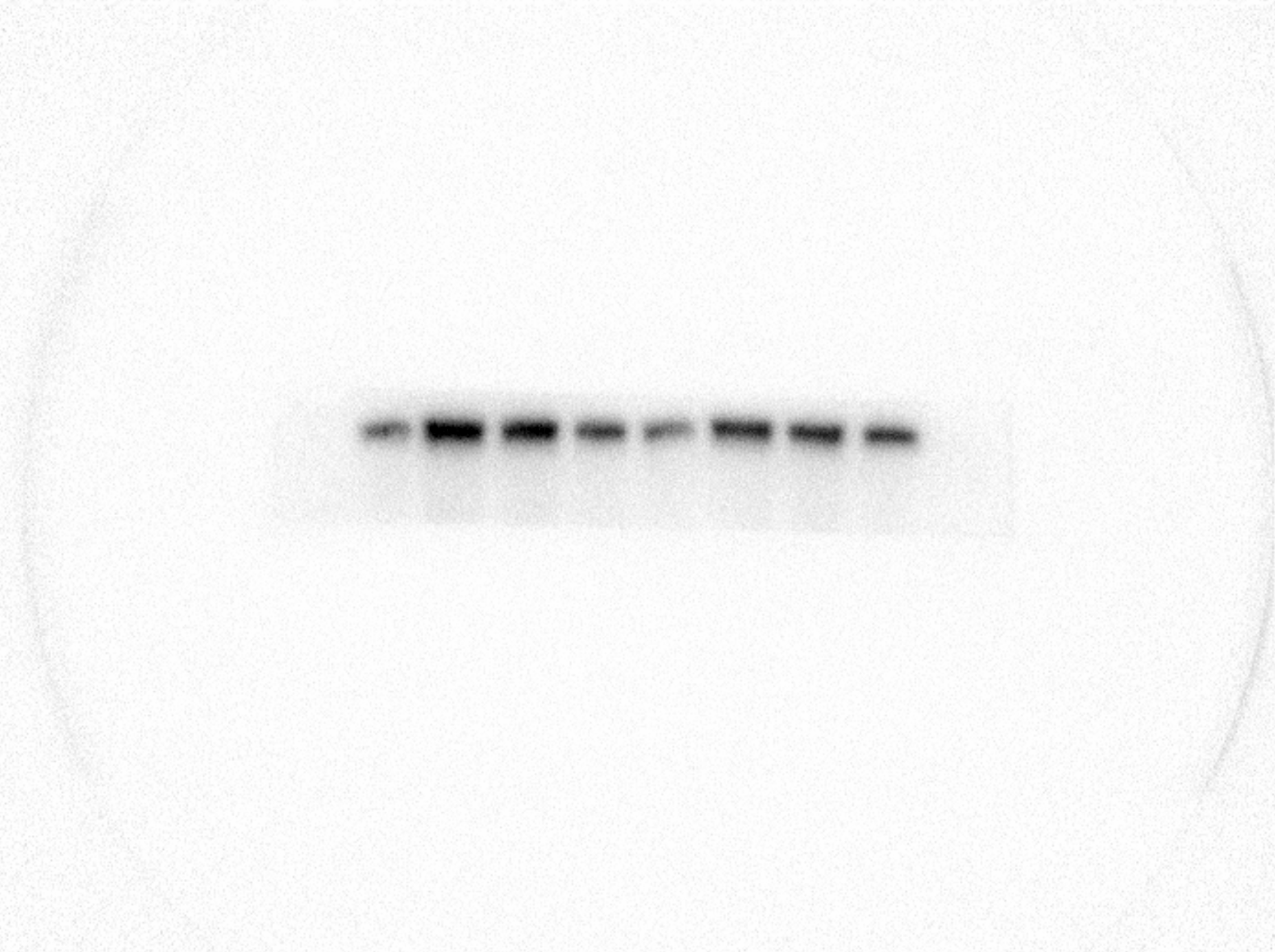

Supplement: Figure 6—figure supplement 1—source data 2. [file elife-73614-fig6-figsupp1-data2.zip › Figure 6S gel/pSmad 2 Gray.tif]

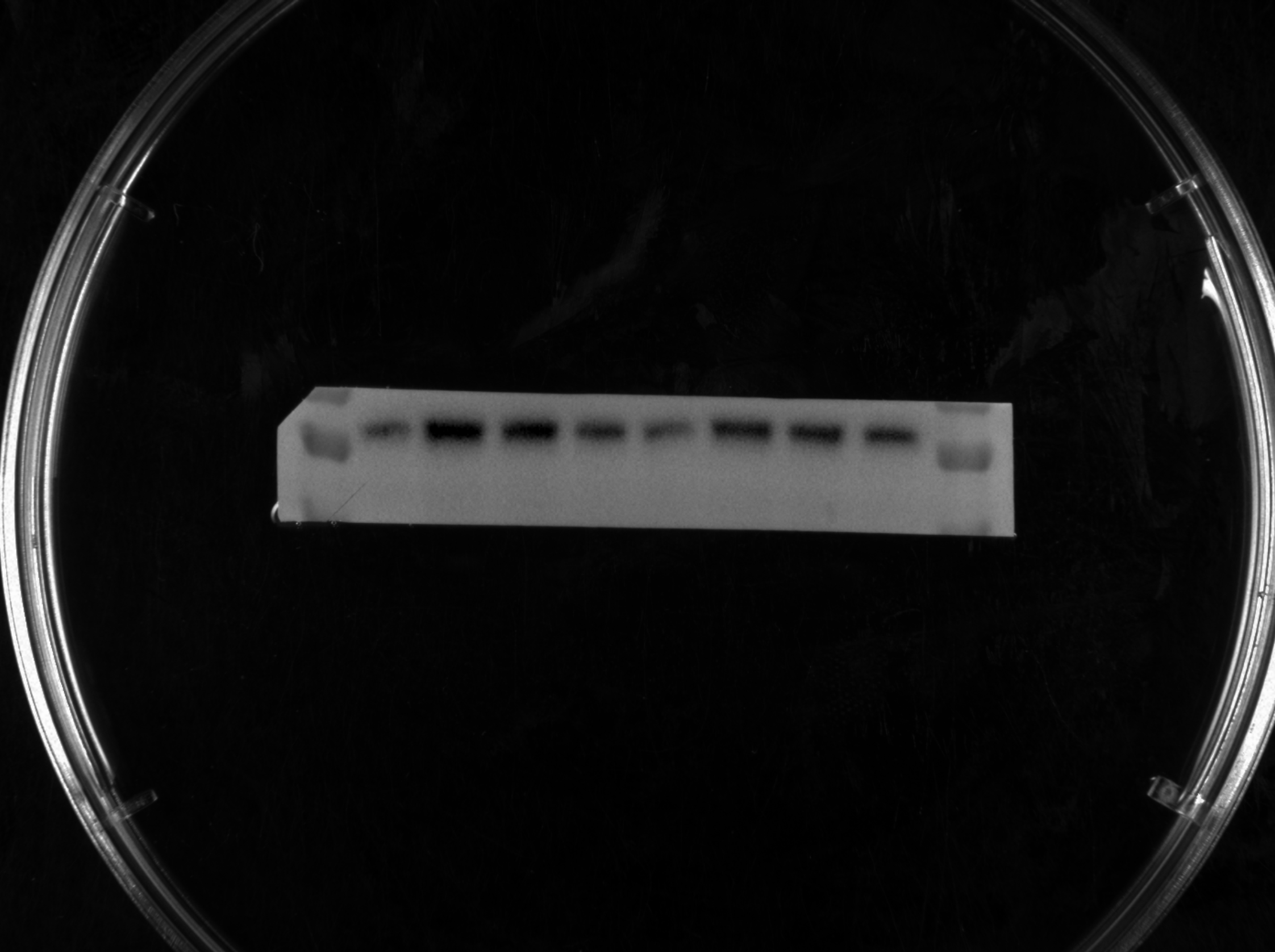

Supplement: Figure 6—figure supplement 1—source data 2. [file elife-73614-fig6-figsupp1-data2.zip › Figure 6S gel/pSmad 2.tif]

## Slide 1
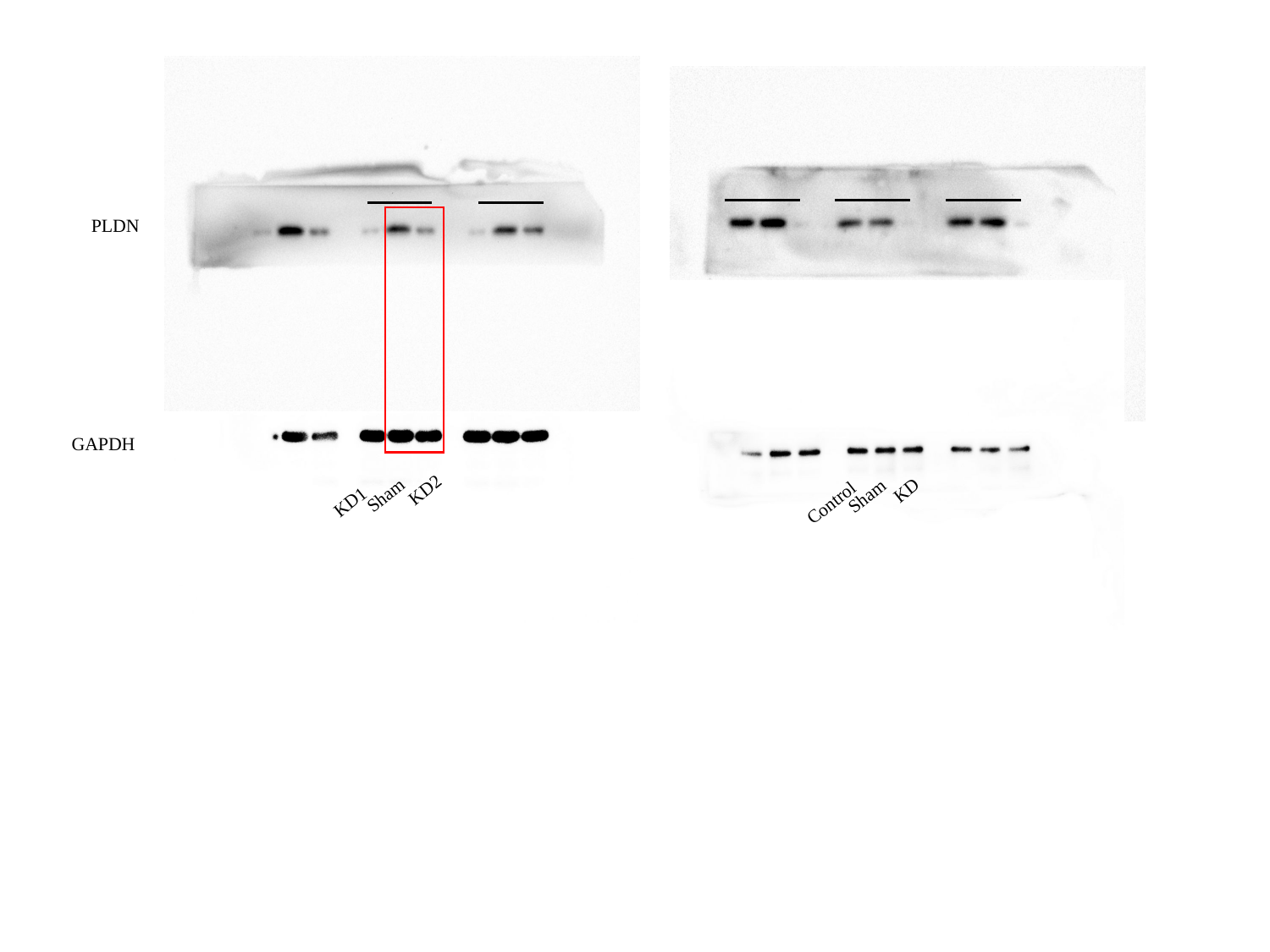

PLDN
GAPDH
KD2
KD
Sham
Sham
KD1
Control

Supplement: Figure 8—source data 2. [file elife-73614-fig8-data2.zip › Figure 8 Source data 2/Figure 8c Source data/Figure 8c Source data.pptx]

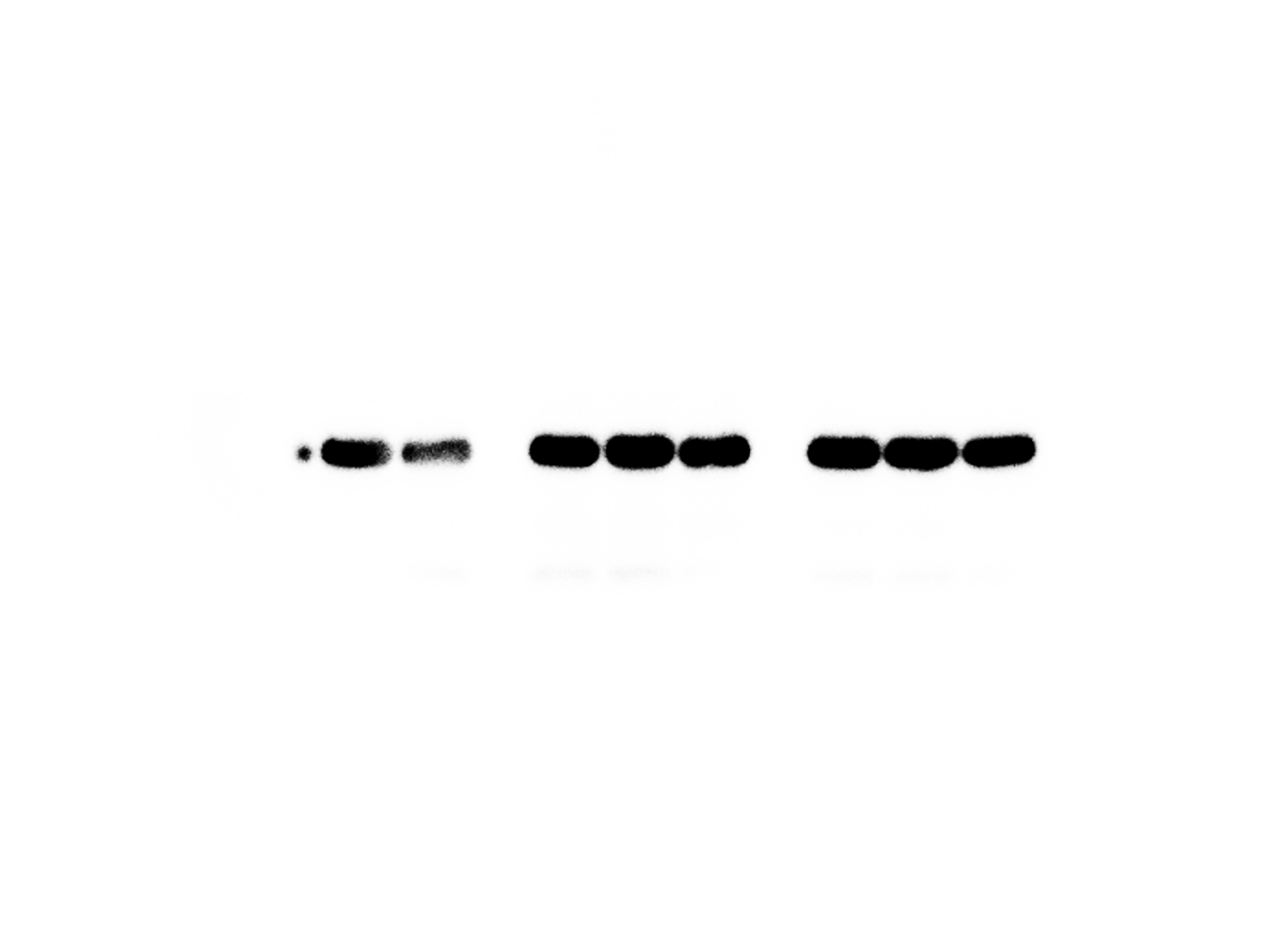

Supplement: Figure 8—source data 2. [file elife-73614-fig8-data2.zip › Figure 8 Source data 2/Figure 8c Source data/GAPDH-1 Gray.tif]

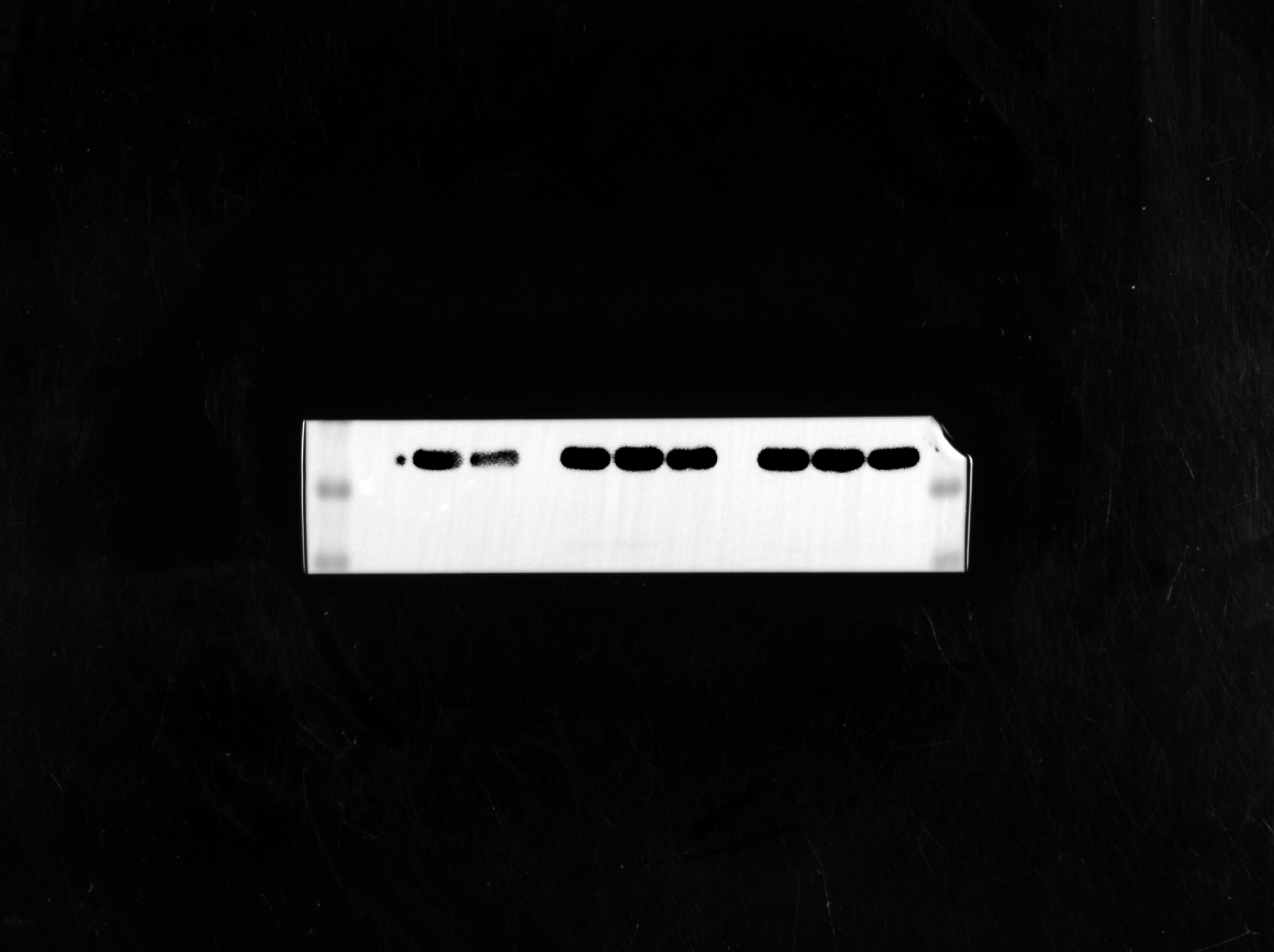

Supplement: Figure 8—source data 2. [file elife-73614-fig8-data2.zip › Figure 8 Source data 2/Figure 8c Source data/GAPDH-1.tif]

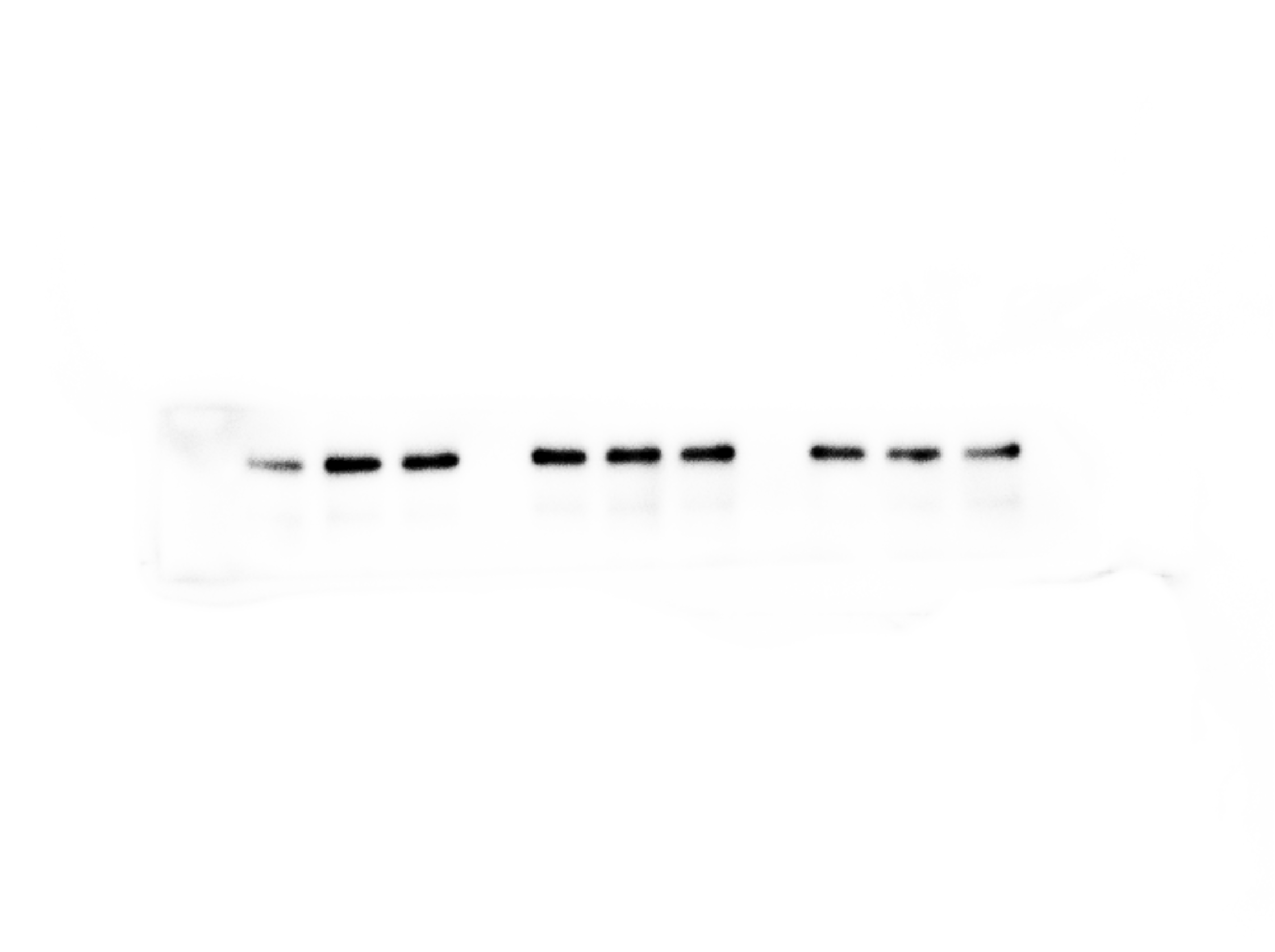

Supplement: Figure 8—source data 2. [file elife-73614-fig8-data2.zip › Figure 8 Source data 2/Figure 8c Source data/GAPDH-2 Gray.tif]

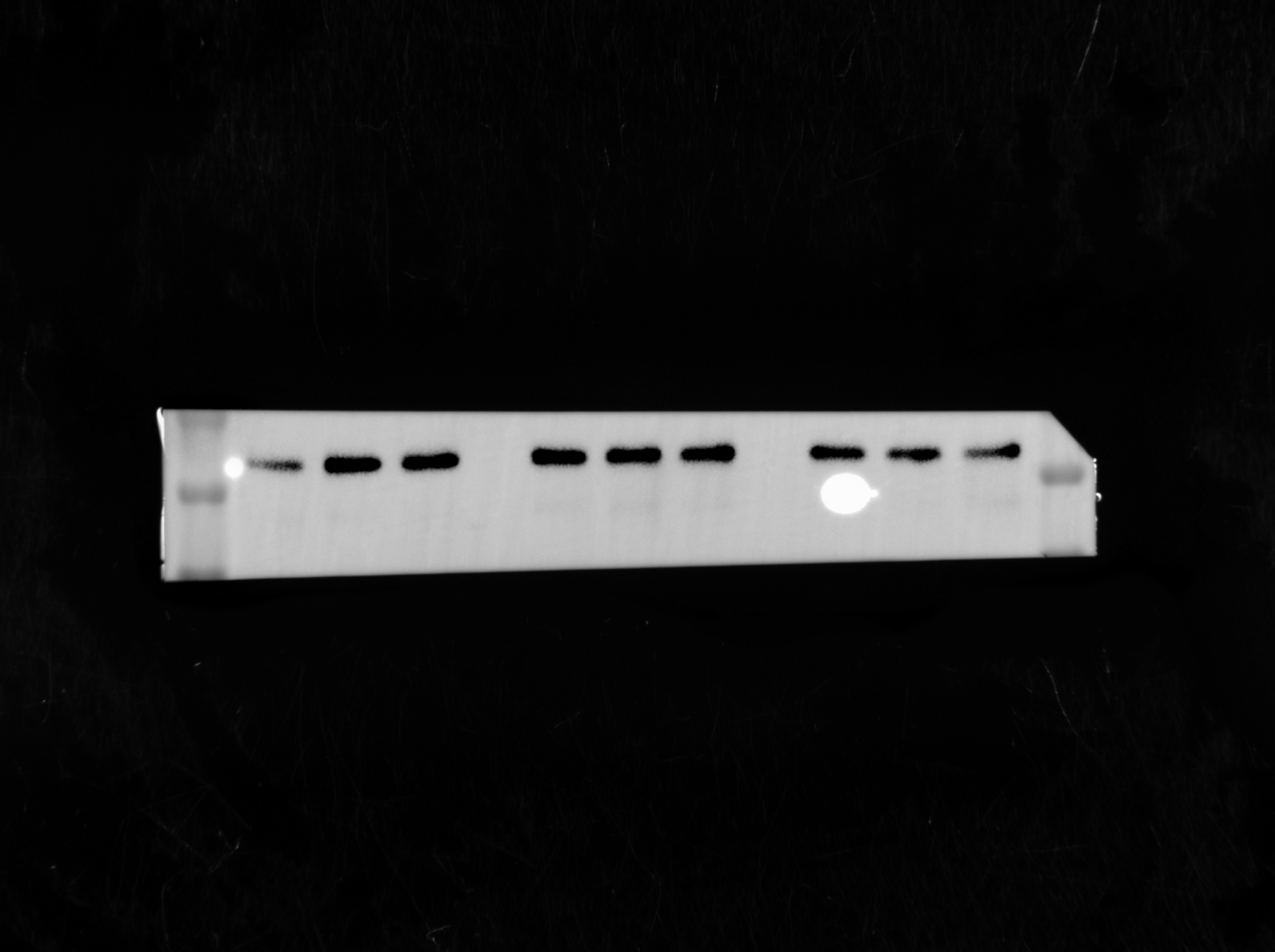

Supplement: Figure 8—source data 2. [file elife-73614-fig8-data2.zip › Figure 8 Source data 2/Figure 8c Source data/GAPDH-2.tif]

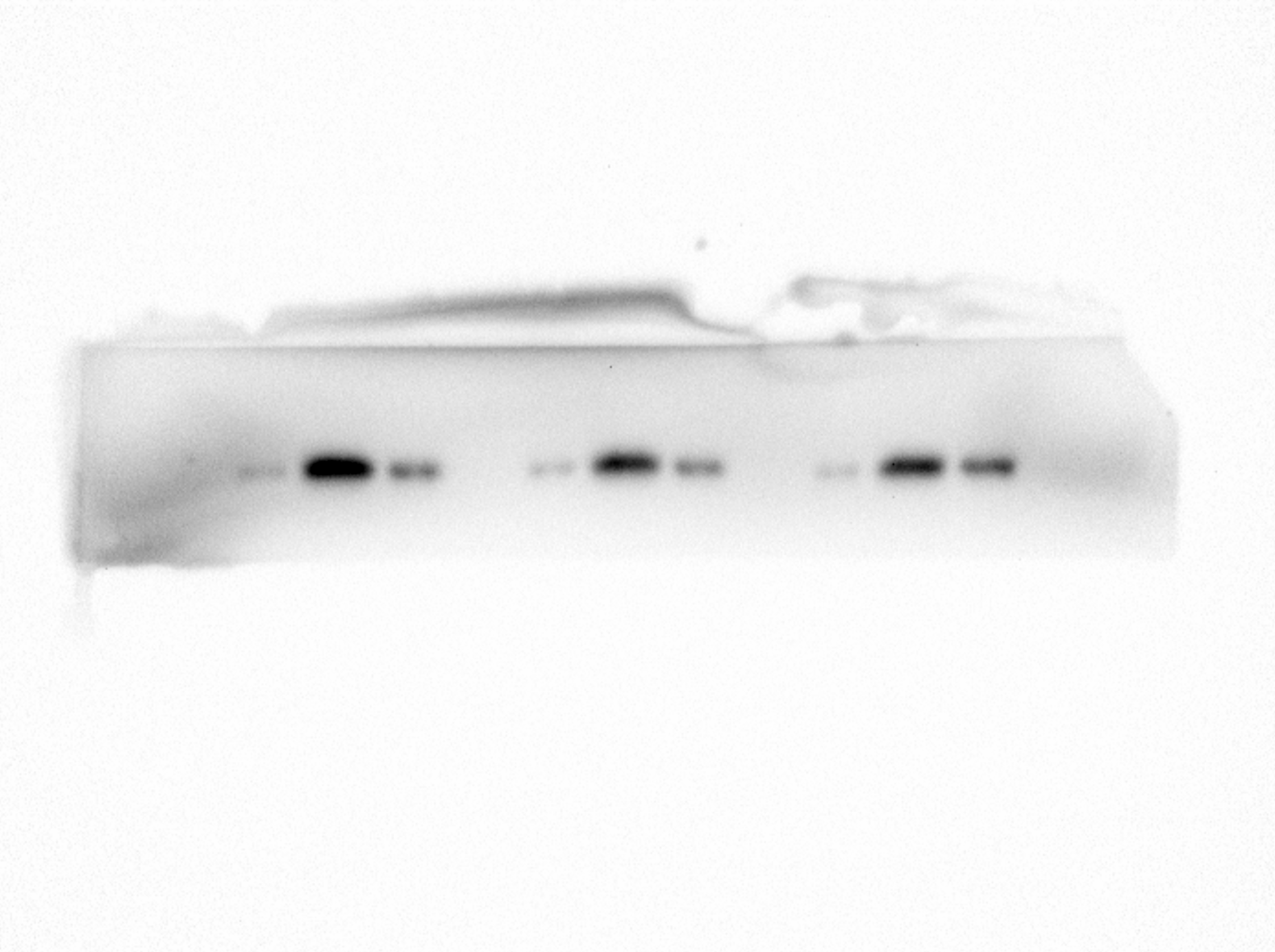

Supplement: Figure 8—source data 2. [file elife-73614-fig8-data2.zip › Figure 8 Source data 2/Figure 8c Source data/PLDN-1 Gray.tif]

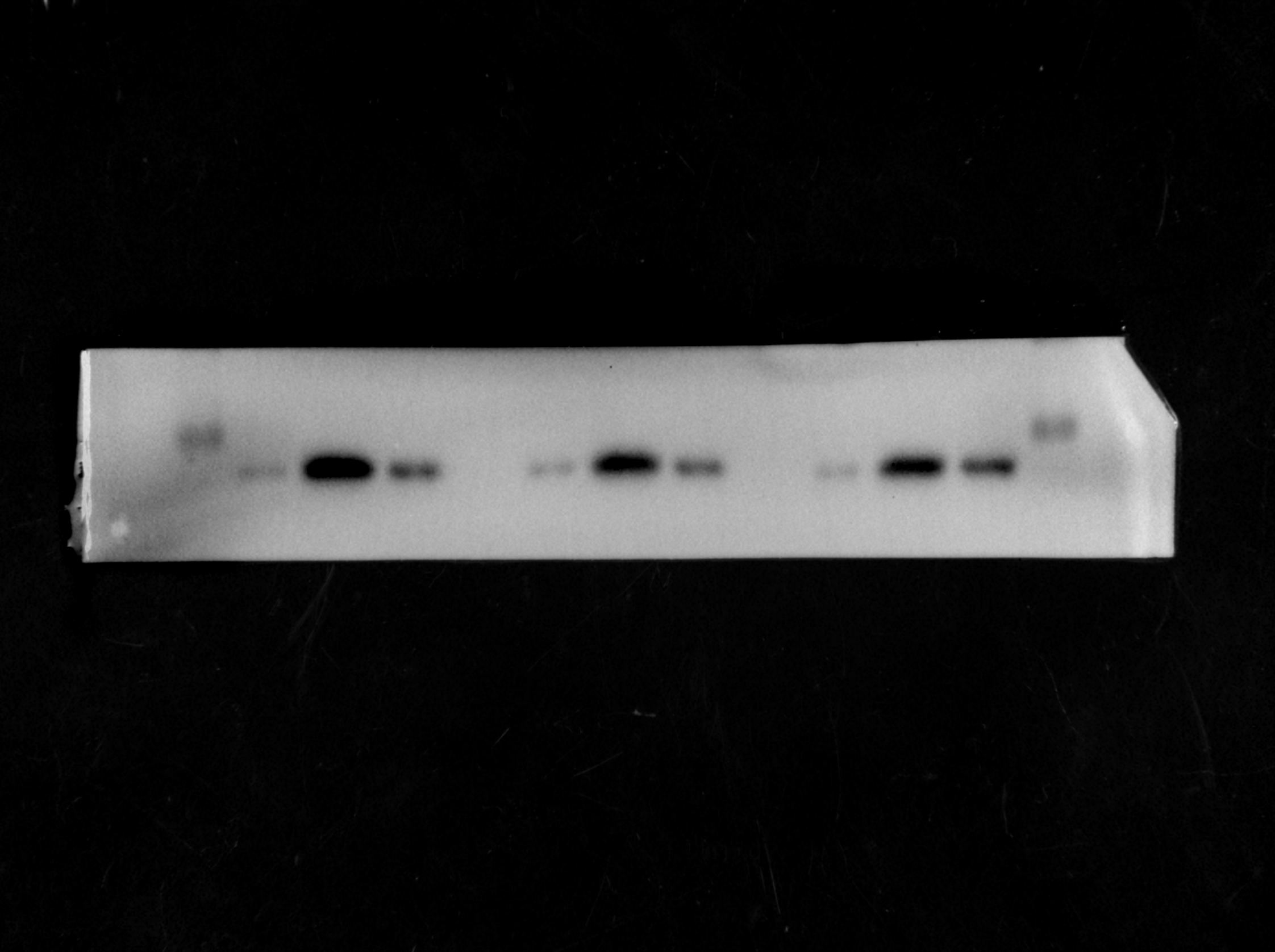

Supplement: Figure 8—source data 2. [file elife-73614-fig8-data2.zip › Figure 8 Source data 2/Figure 8c Source data/PLDN-1.tif]

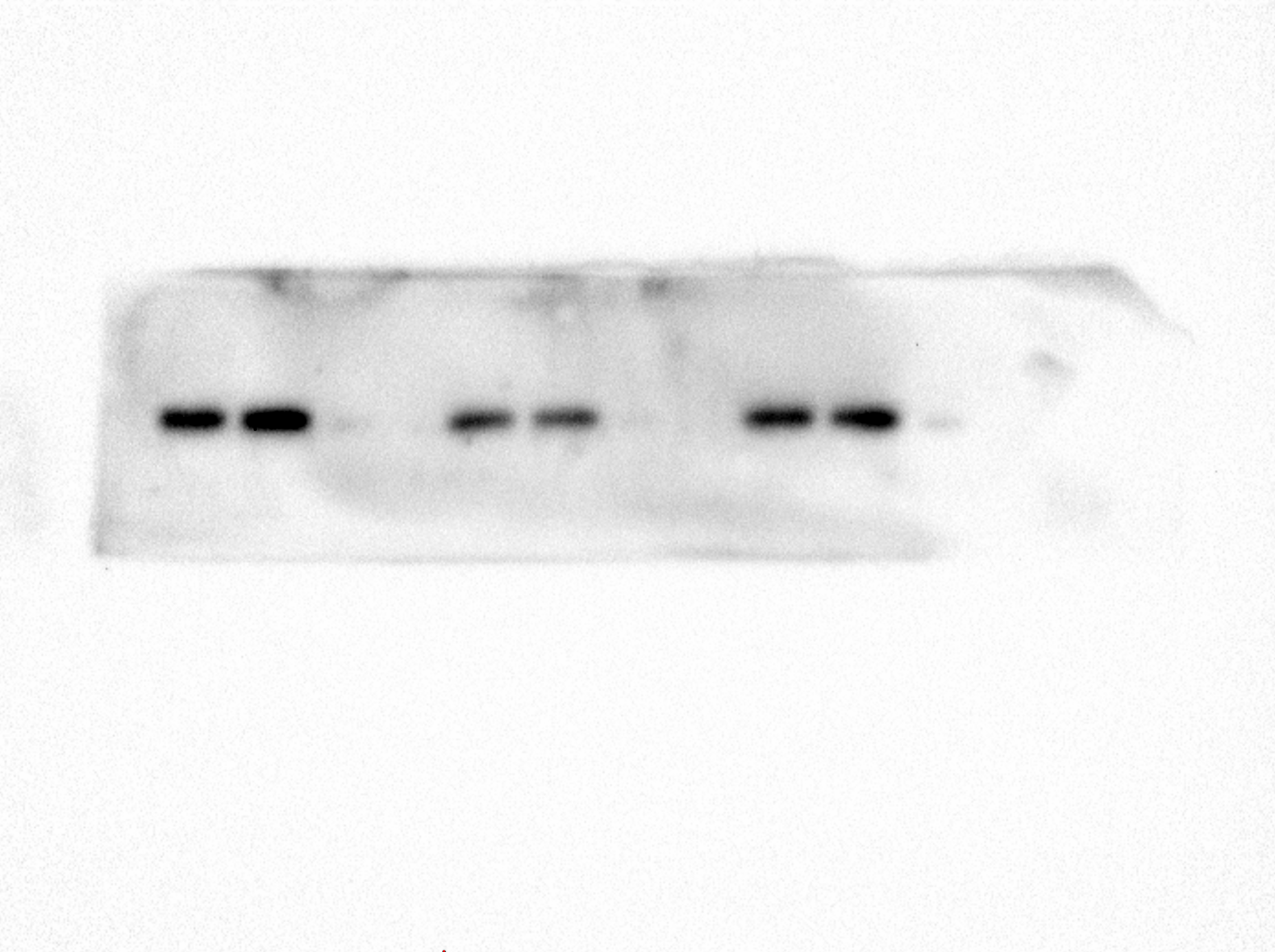

Supplement: Figure 8—source data 2. [file elife-73614-fig8-data2.zip › Figure 8 Source data 2/Figure 8c Source data/PLDN-2 Gray.tif]

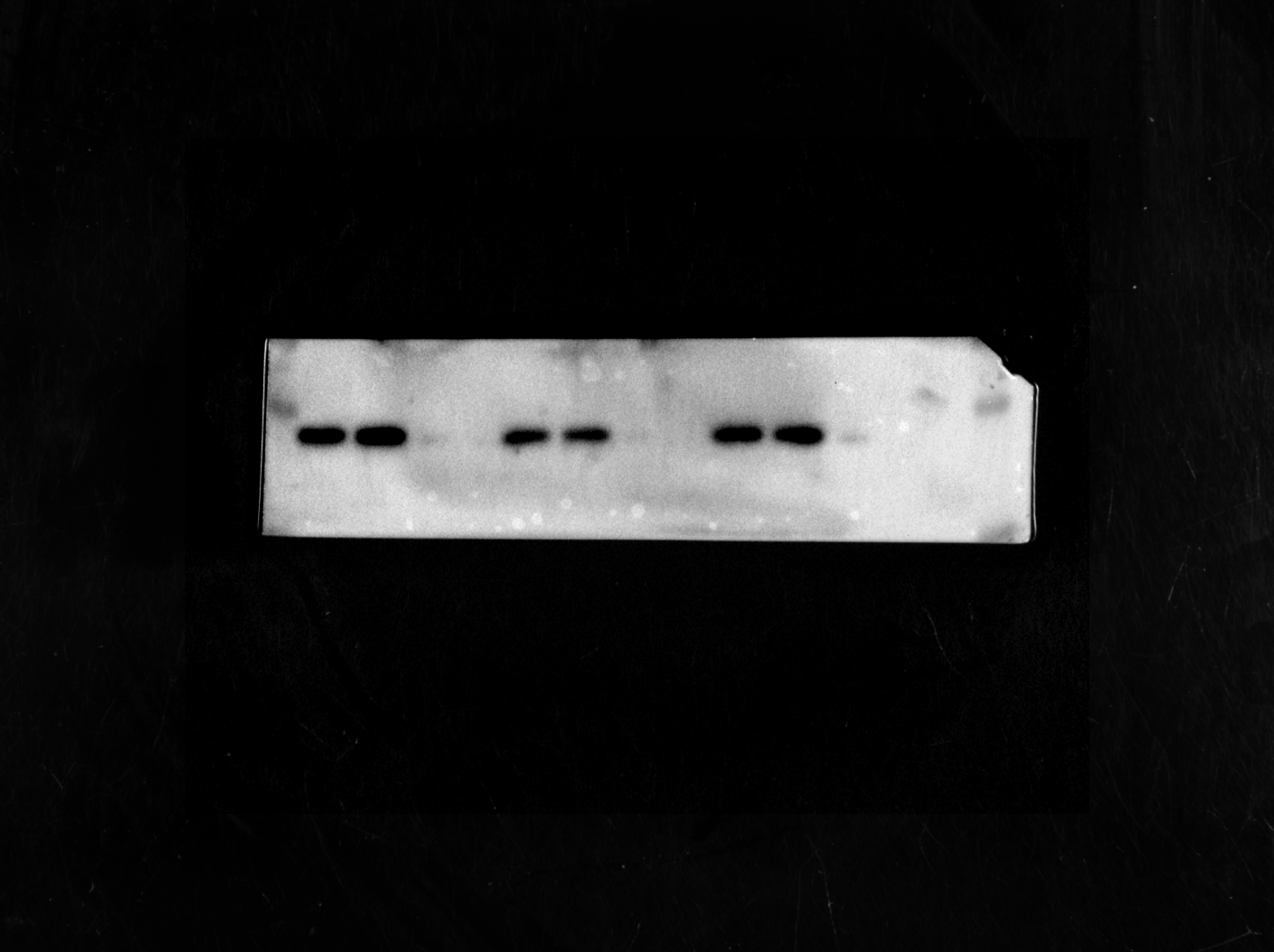

Supplement: Figure 8—source data 2. [file elife-73614-fig8-data2.zip › Figure 8 Source data 2/Figure 8c Source data/PLDN-2.tif]

## Slide 1
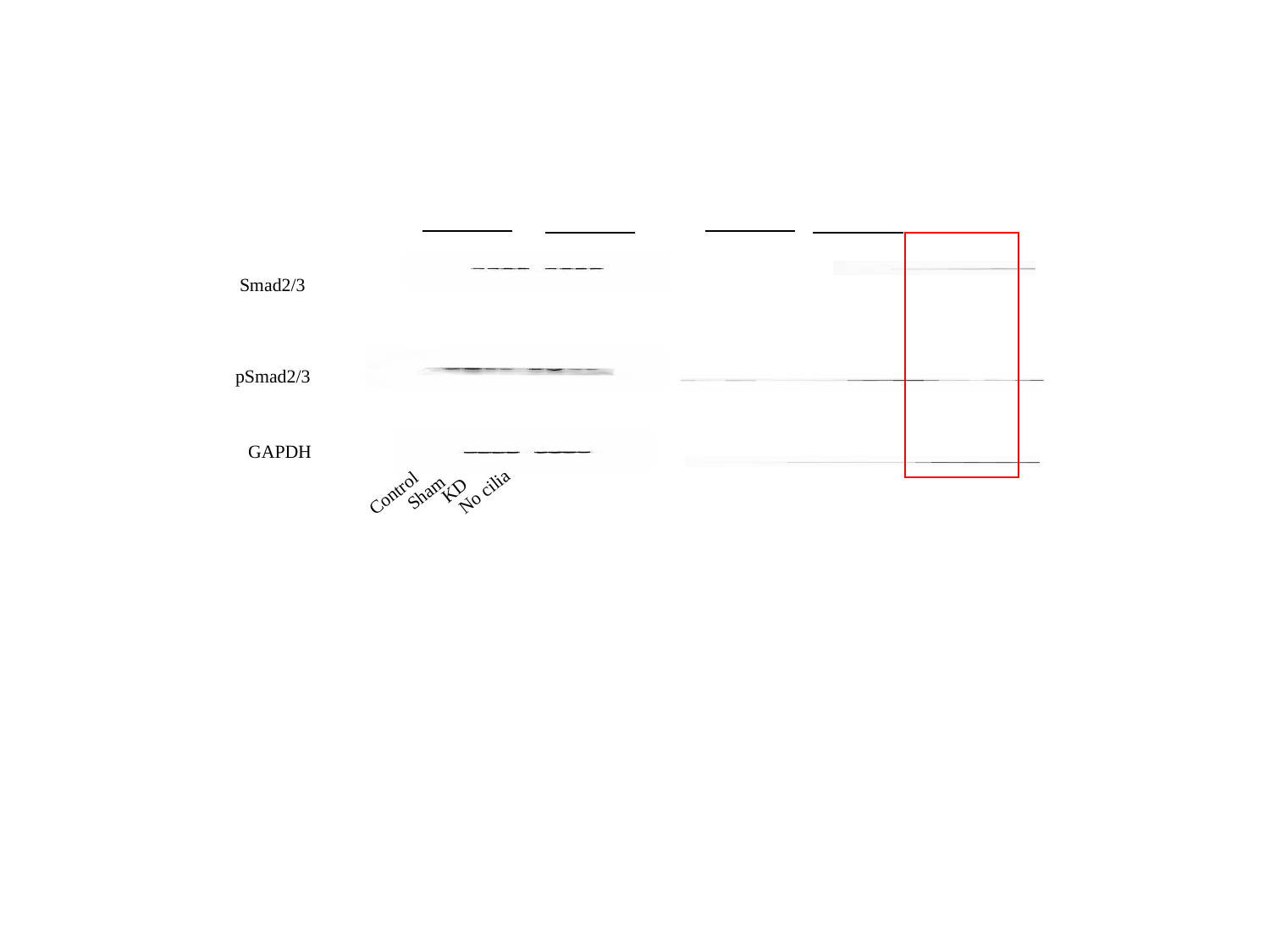

Smad2/3
pSmad2/3
GAPDH
KD
No cilia
Sham
Control

Supplement: Figure 8—source data 2. [file elife-73614-fig8-data2.zip › Figure 8 Source data 2/Figure 8j Source data/Figure 8j Source data.pptx]

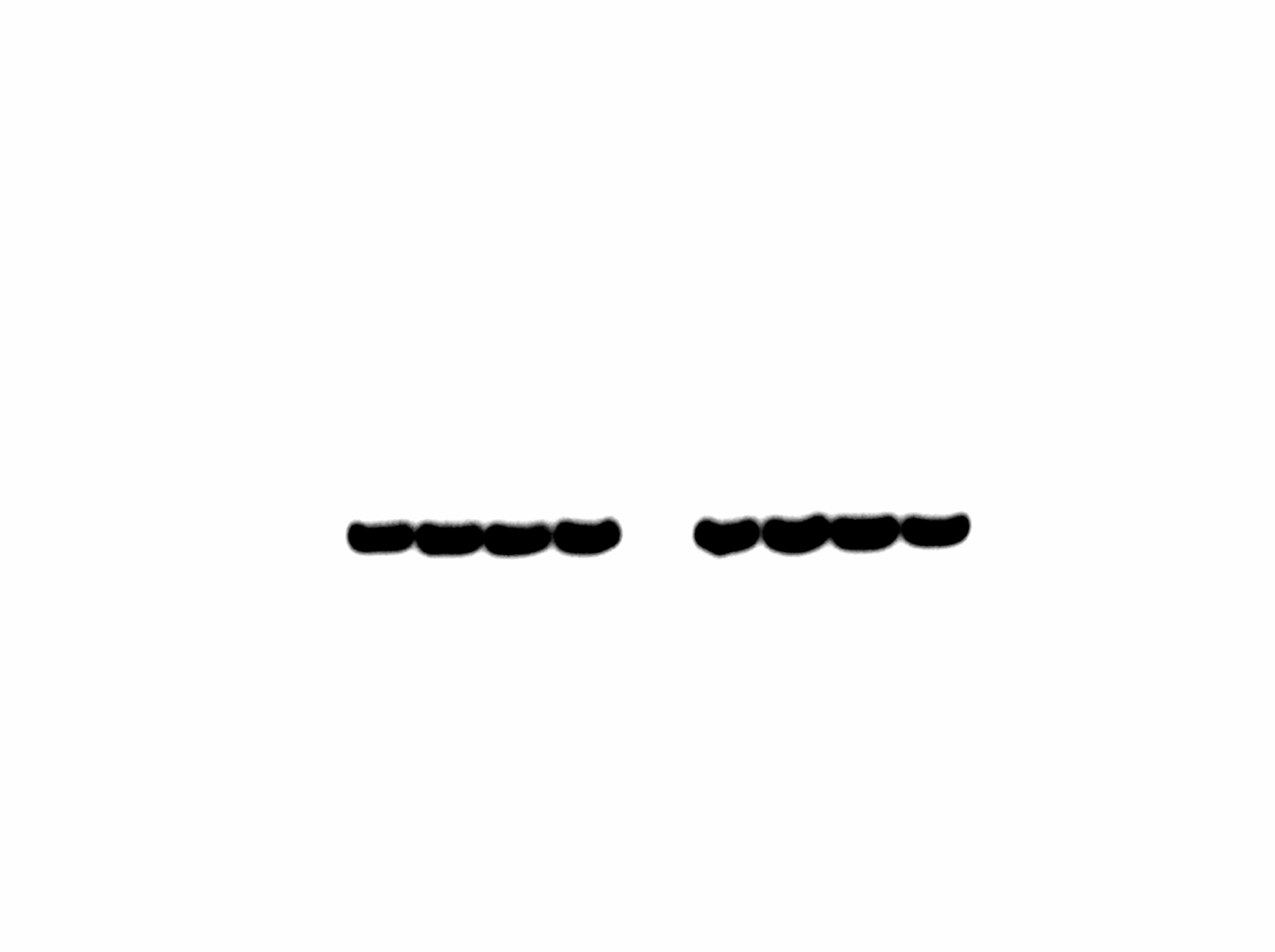

Supplement: Figure 8—source data 2. [file elife-73614-fig8-data2.zip › Figure 8 Source data 2/Figure 8j Source data/GAPDH 1 Gray.tif]

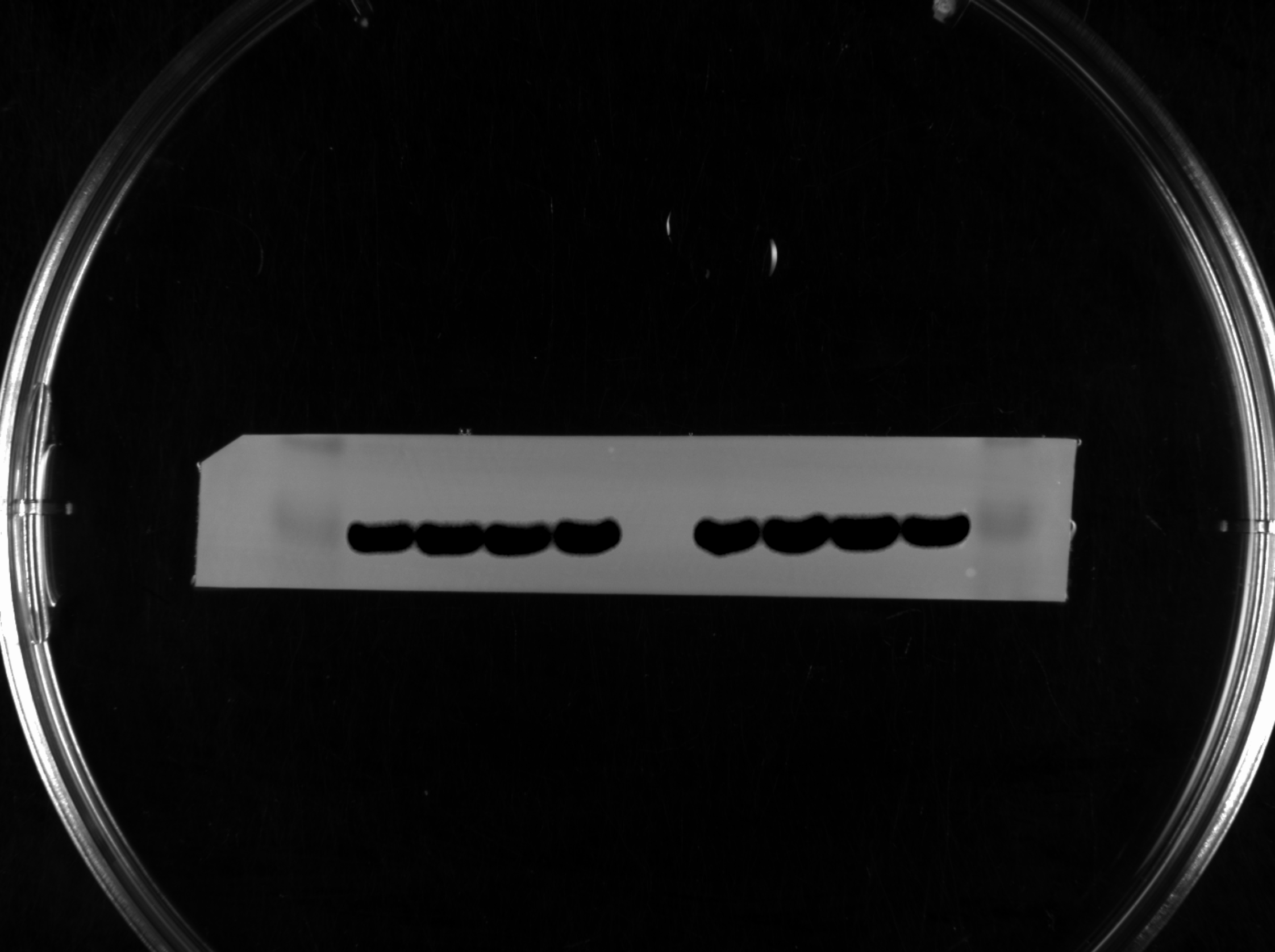

Supplement: Figure 8—source data 2. [file elife-73614-fig8-data2.zip › Figure 8 Source data 2/Figure 8j Source data/GAPDH 1.tif]

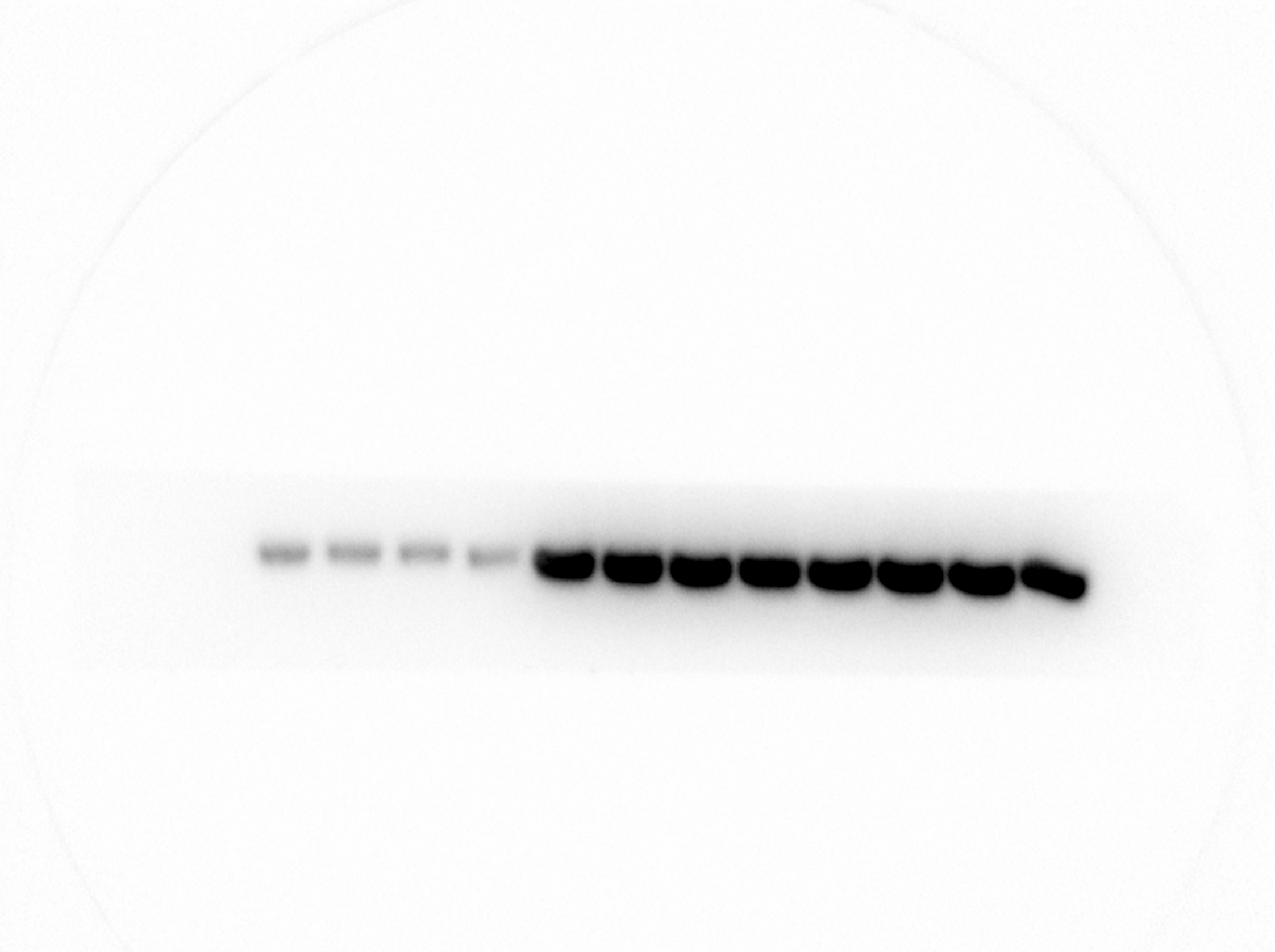

Supplement: Figure 8—source data 2. [file elife-73614-fig8-data2.zip › Figure 8 Source data 2/Figure 8j Source data/GAPDH 2 Gray.tif]

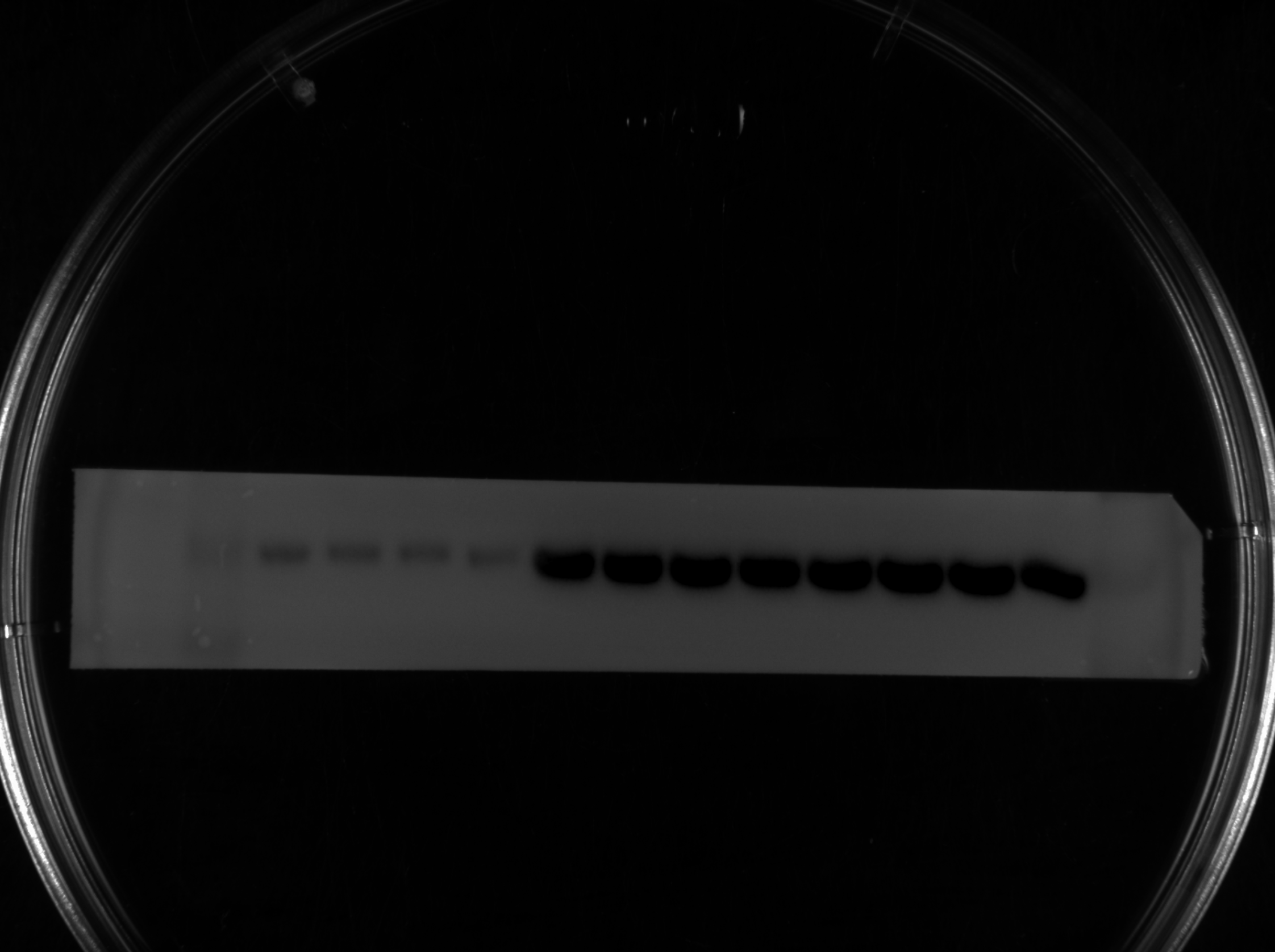

Supplement: Figure 8—source data 2. [file elife-73614-fig8-data2.zip › Figure 8 Source data 2/Figure 8j Source data/GAPDH 2.tif]

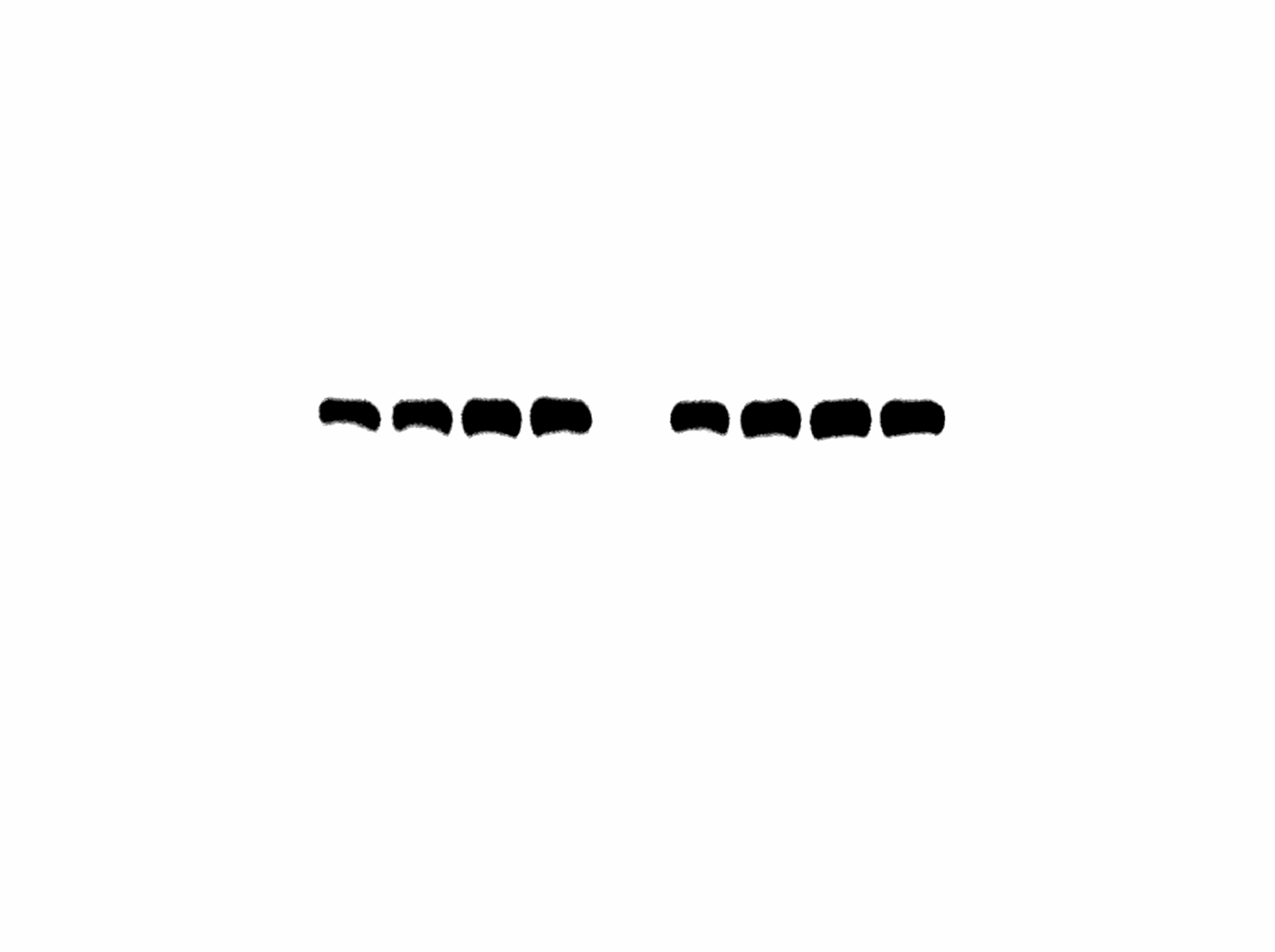

Supplement: Figure 8—source data 2. [file elife-73614-fig8-data2.zip › Figure 8 Source data 2/Figure 8j Source data/Smad 1 Gray.tif]

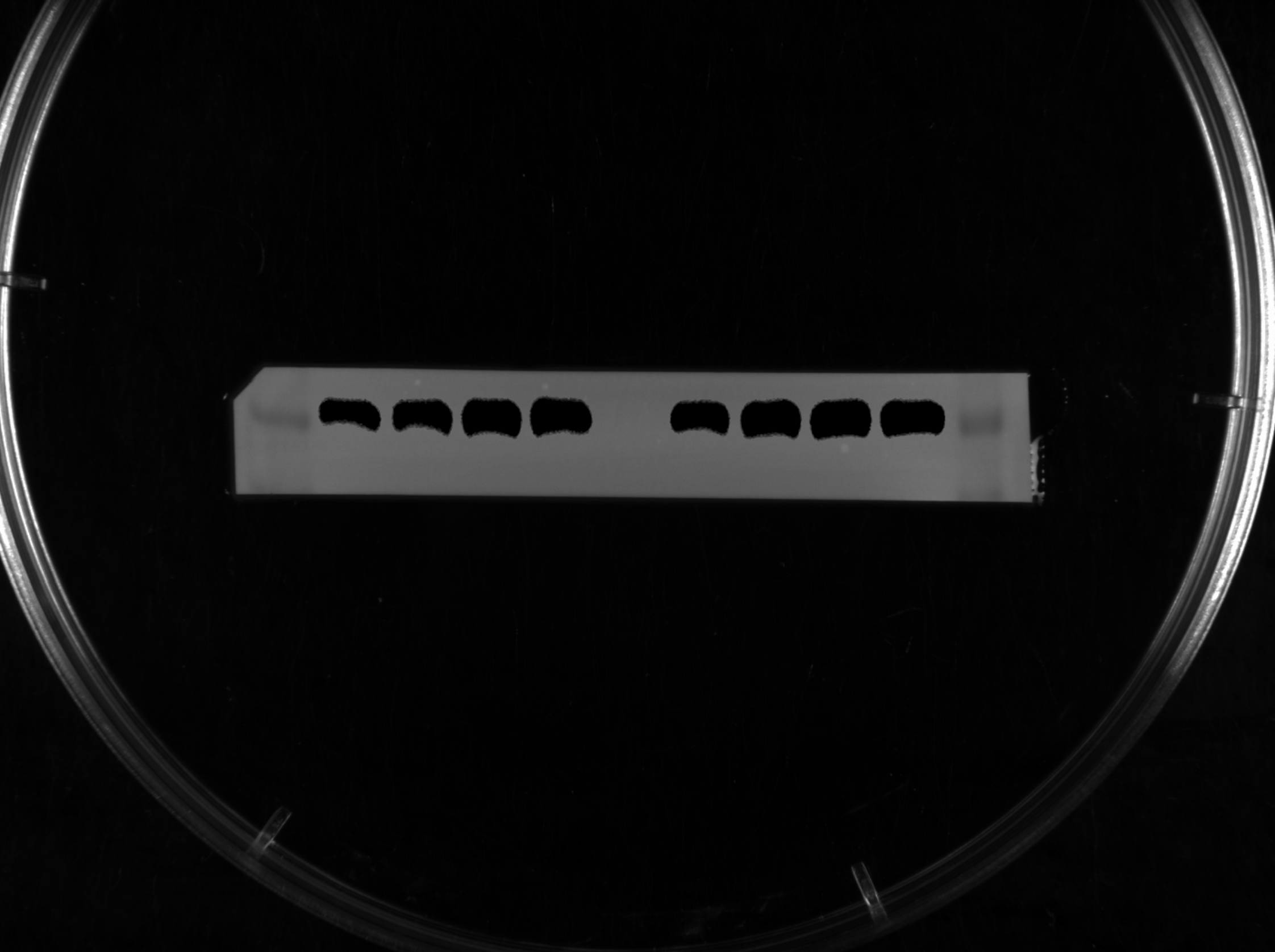

Supplement: Figure 8—source data 2. [file elife-73614-fig8-data2.zip › Figure 8 Source data 2/Figure 8j Source data/Smad 1.tif]

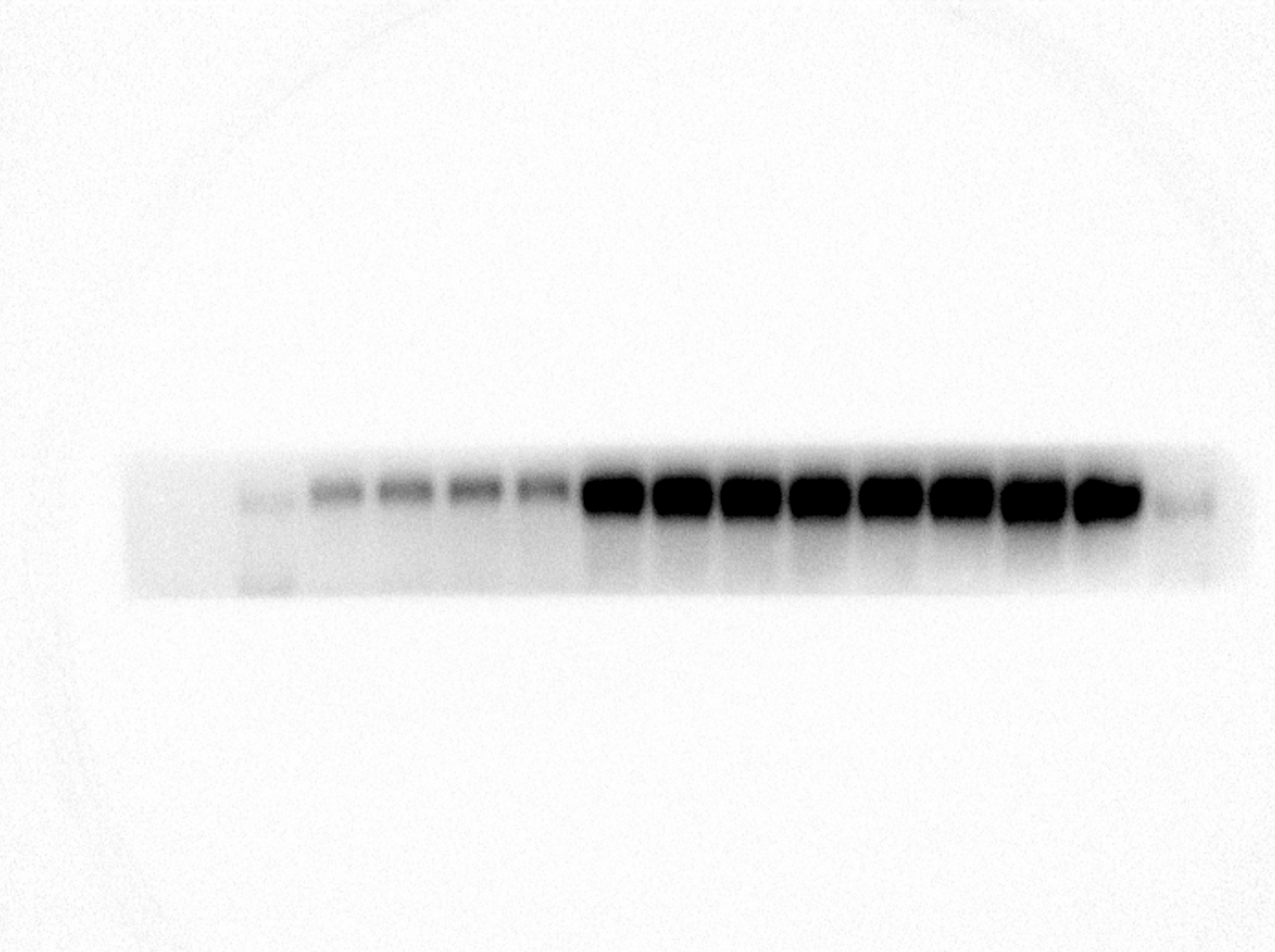

Supplement: Figure 8—source data 2. [file elife-73614-fig8-data2.zip › Figure 8 Source data 2/Figure 8j Source data/Smad 2 Gray.tif]

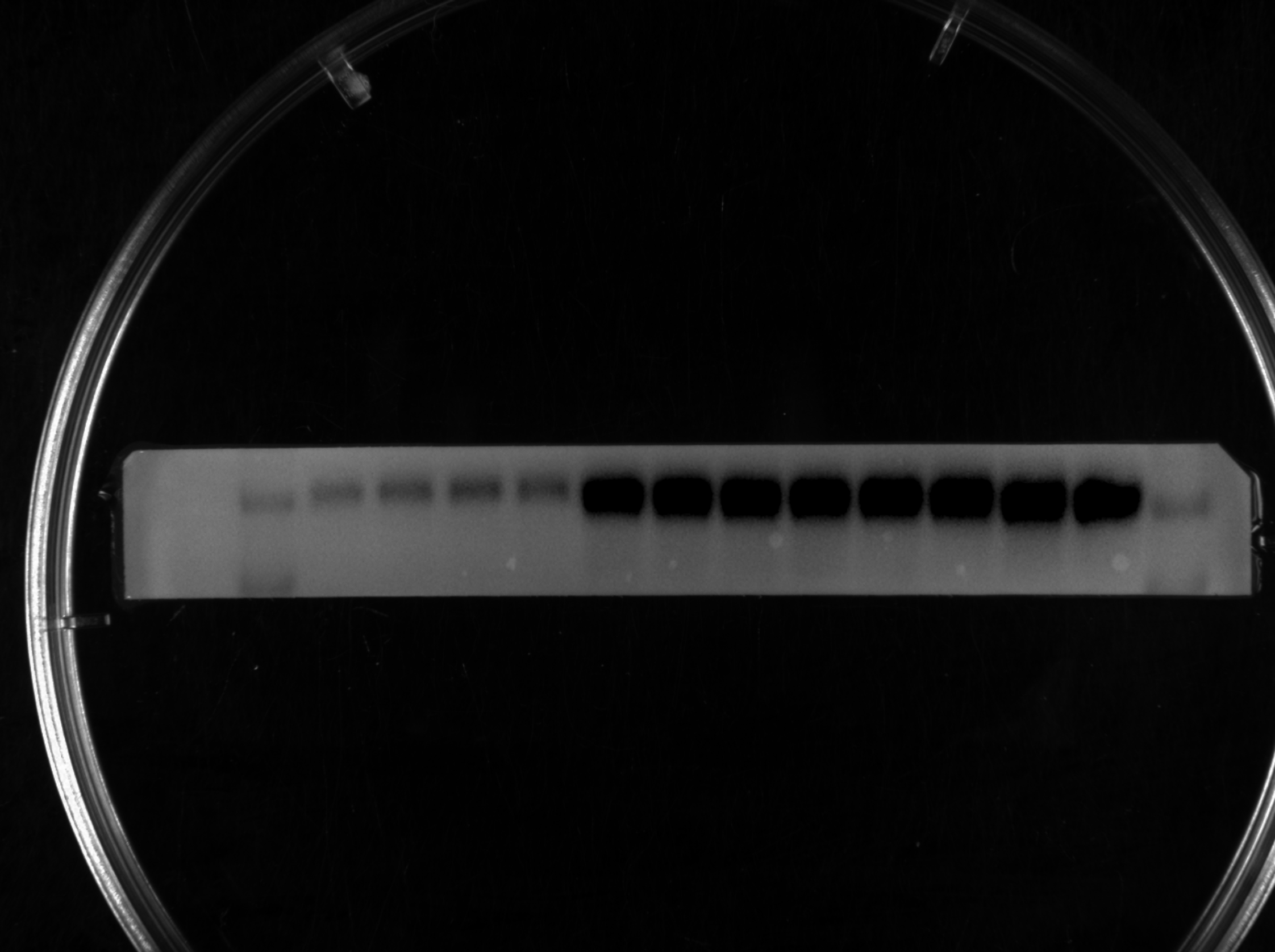

Supplement: Figure 8—source data 2. [file elife-73614-fig8-data2.zip › Figure 8 Source data 2/Figure 8j Source data/Smad 2.tif]

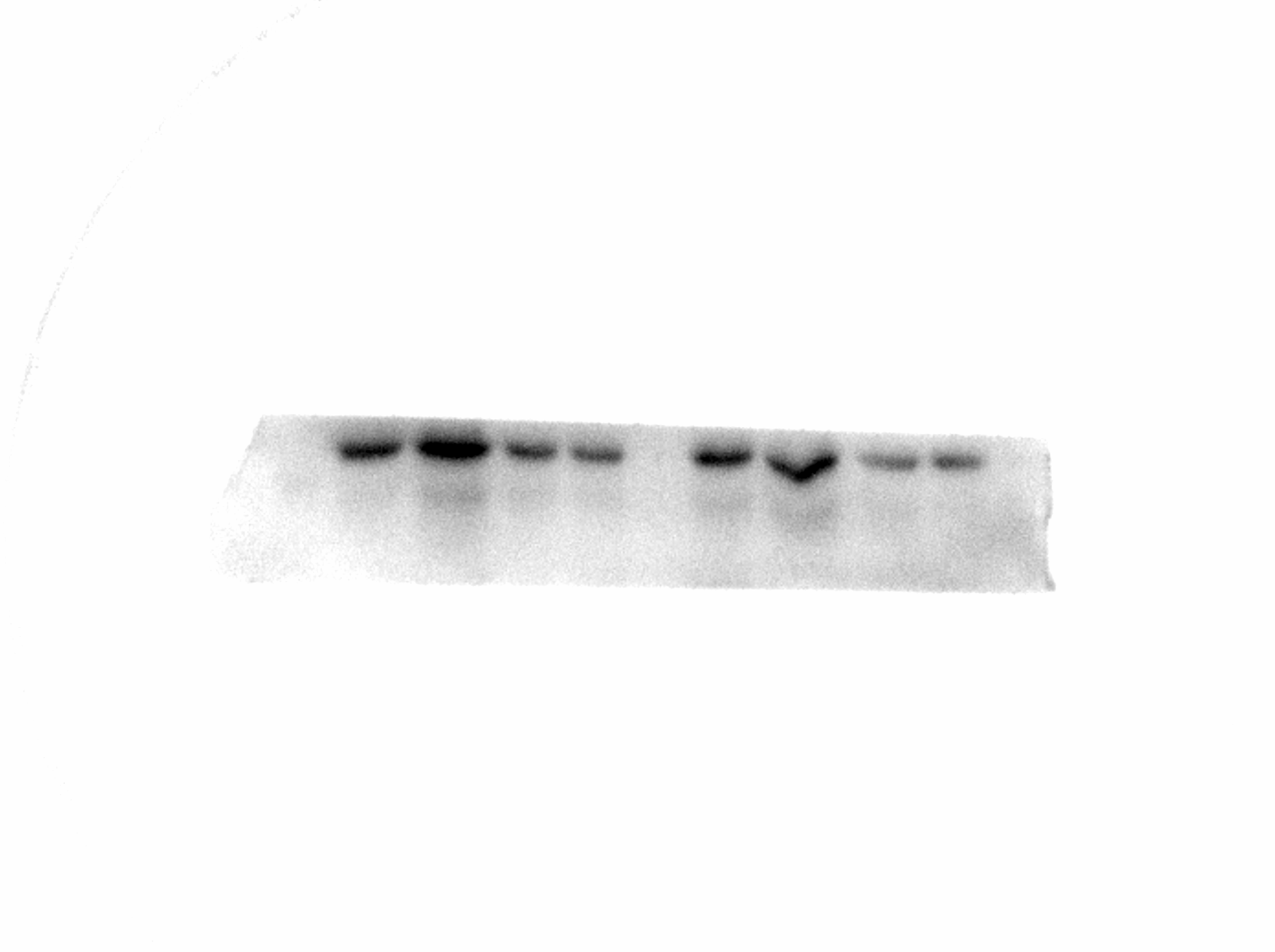

Supplement: Figure 8—source data 2. [file elife-73614-fig8-data2.zip › Figure 8 Source data 2/Figure 8j Source data/pSmad 1 Gray.tif]

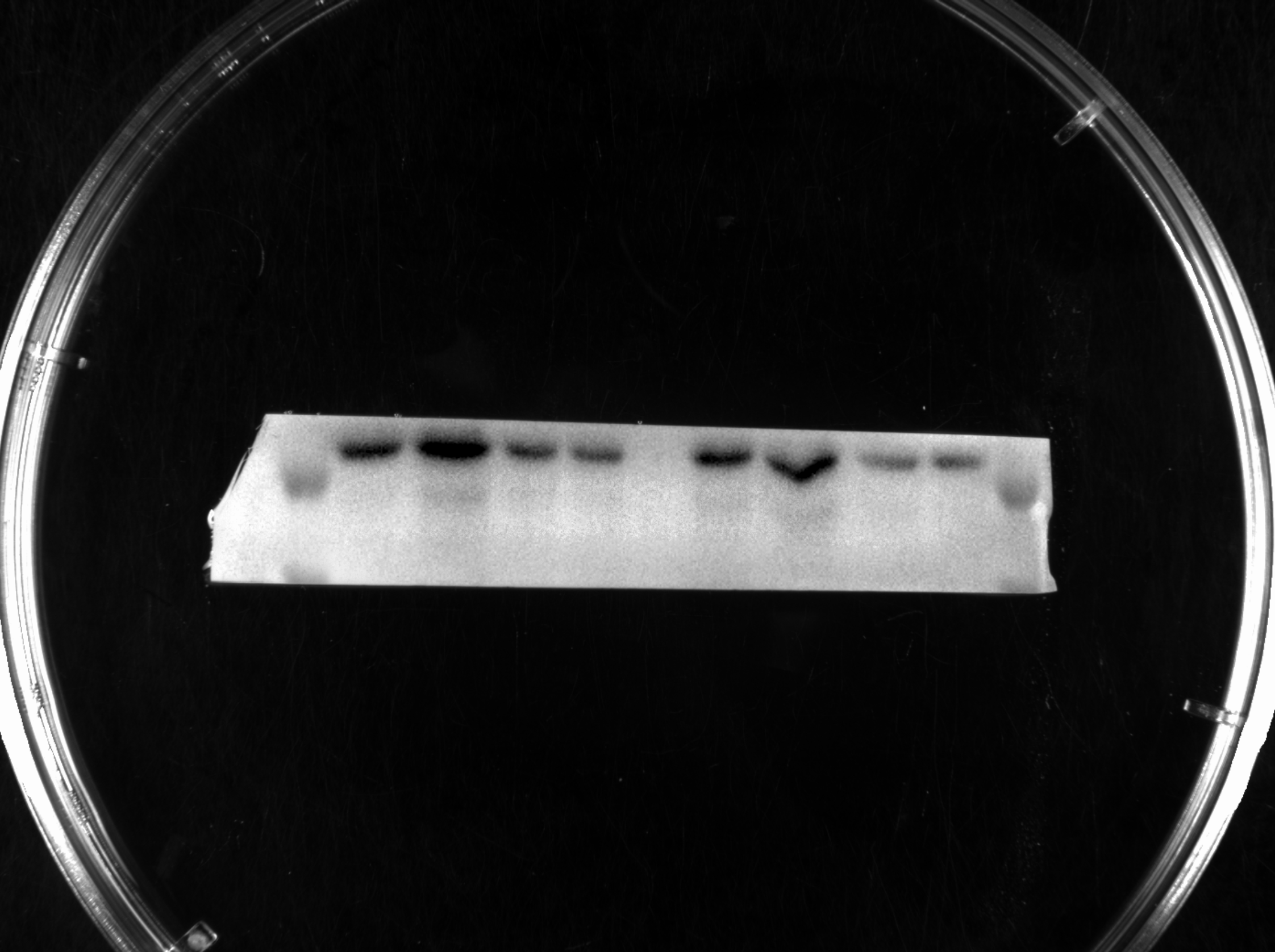

Supplement: Figure 8—source data 2. [file elife-73614-fig8-data2.zip › Figure 8 Source data 2/Figure 8j Source data/pSmad 1.tif]

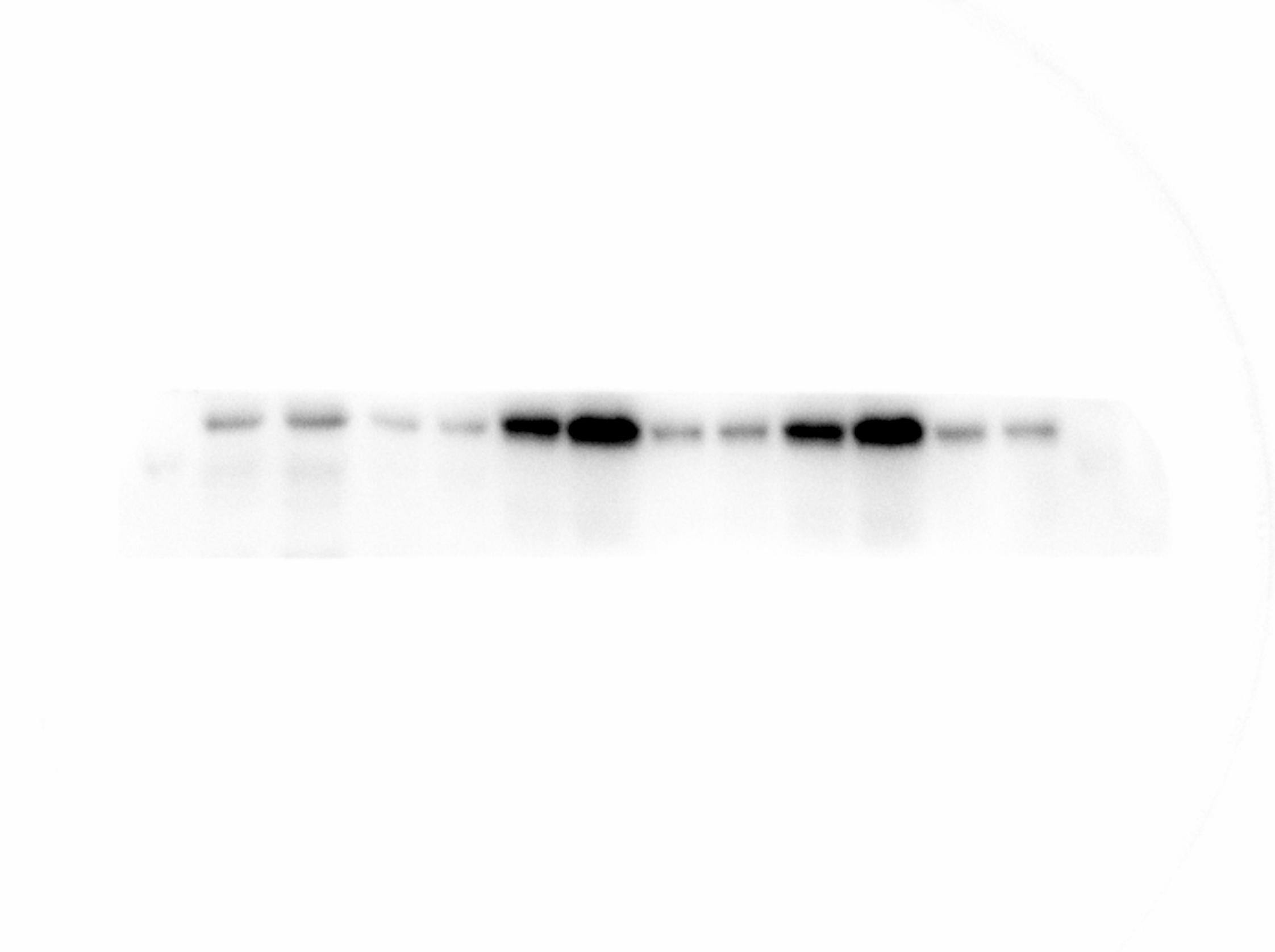

Supplement: Figure 8—source data 2. [file elife-73614-fig8-data2.zip › Figure 8 Source data 2/Figure 8j Source data/pSmad 2 Gray.tif]

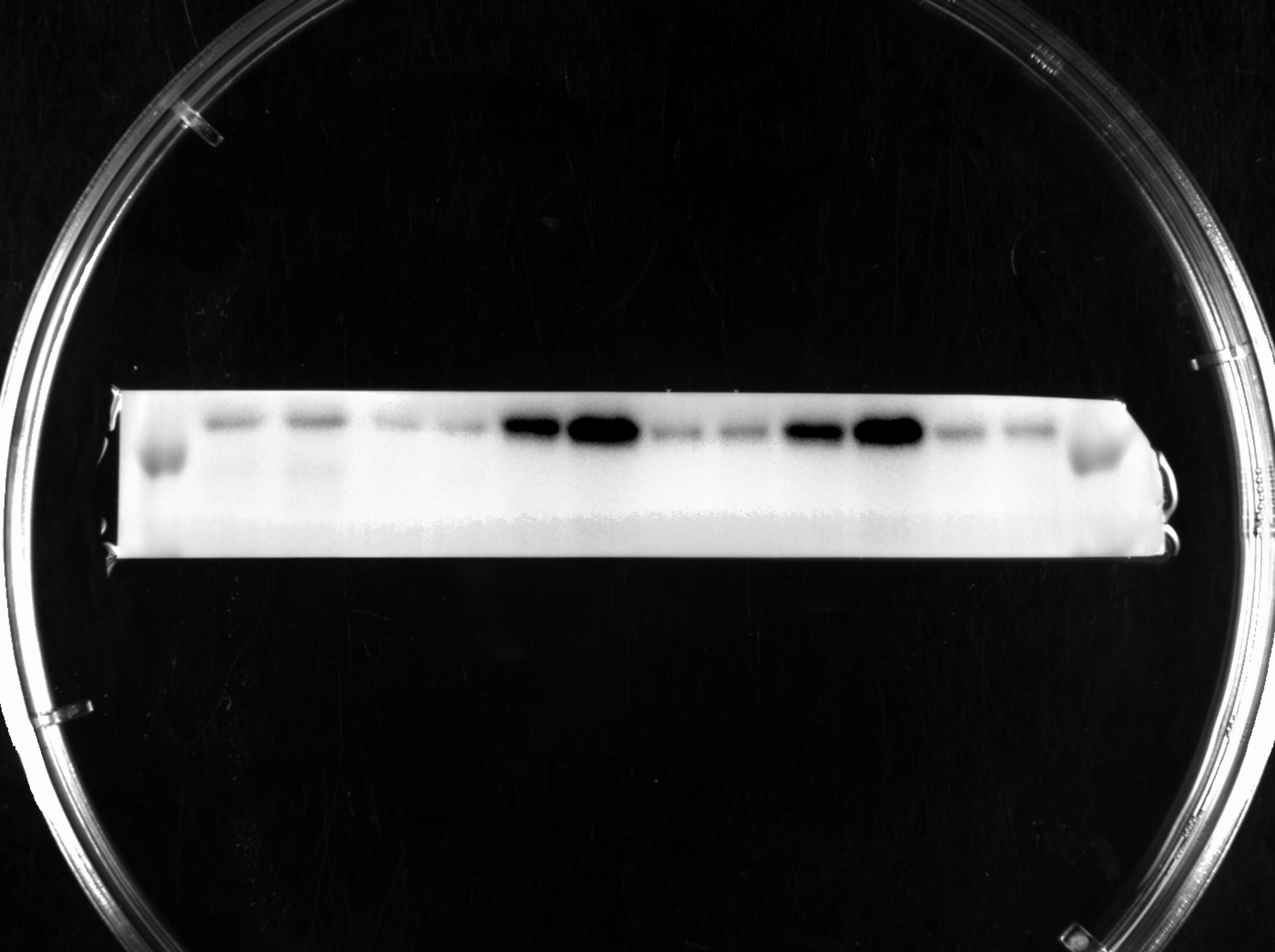

Supplement: Figure 8—source data 2. [file elife-73614-fig8-data2.zip › Figure 8 Source data 2/Figure 8j Source data/pSmad 2.tif]

## Slide 1
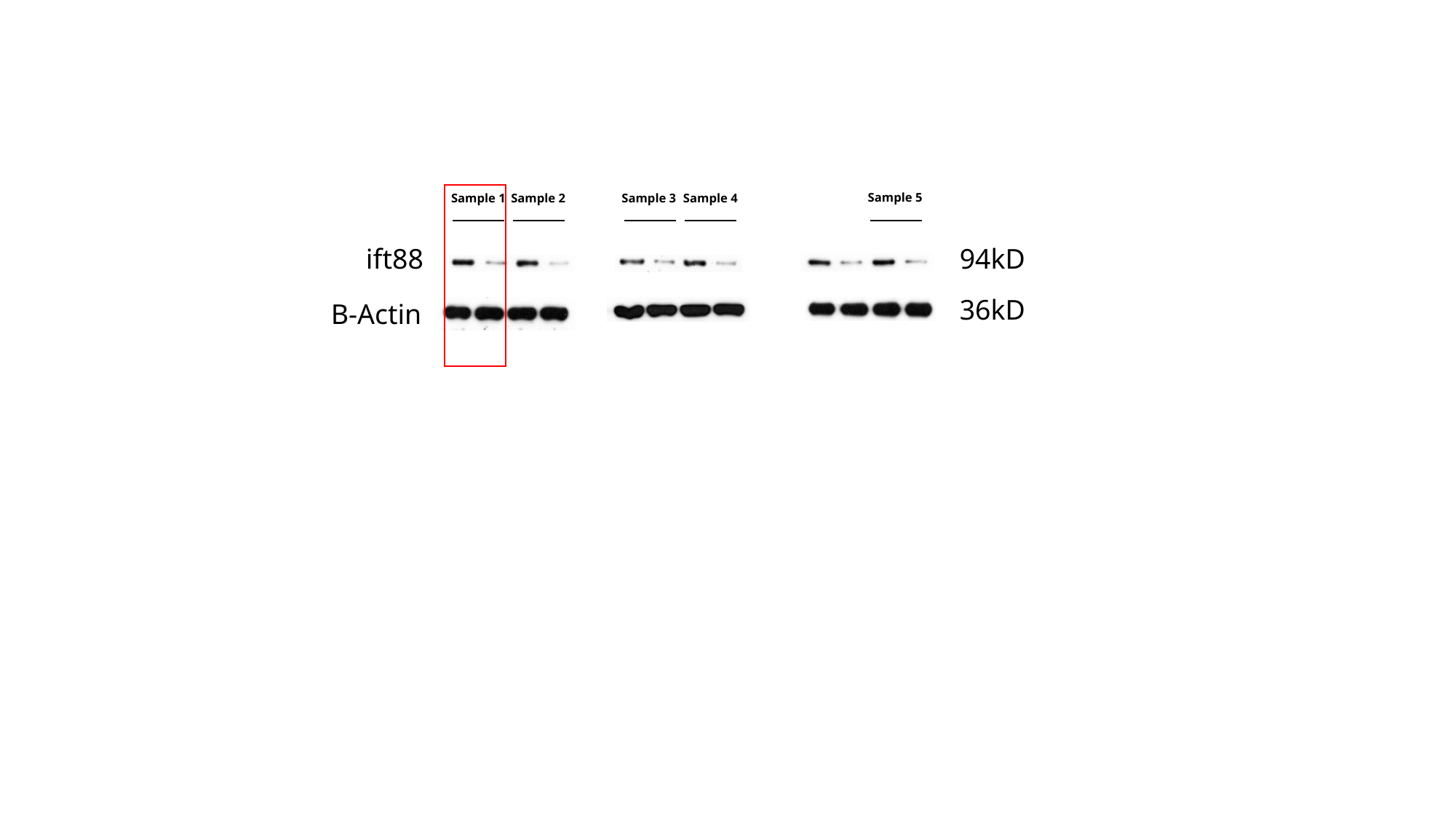

Sample 5
Sample 1
Sample 2
Sample 3
Sample 4
ift88
94kD
36kD
Β-Actin

Supplement: Figure 8—figure supplement 1—source data 2. [file elife-73614-fig8-figsupp1-data2.zip › Figure 8S gel/Figure 8S gel.pptx]

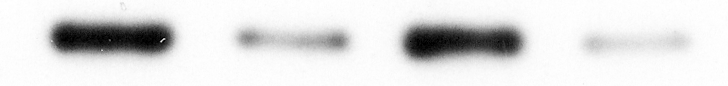

Supplement: Figure 8—figure supplement 1—source data 2. [file elife-73614-fig8-figsupp1-data2.zip › Figure 8S gel/IFT88 1-Gray.tif]

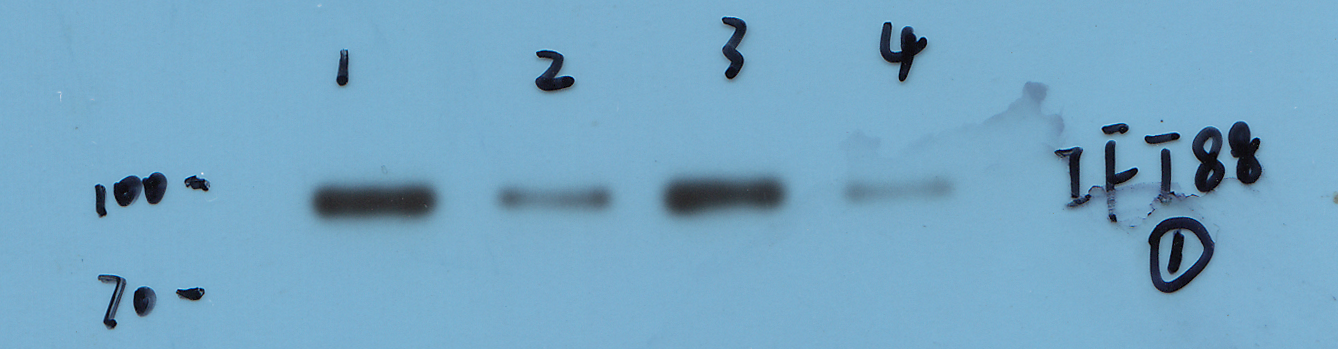

Supplement: Figure 8—figure supplement 1—source data 2. [file elife-73614-fig8-figsupp1-data2.zip › Figure 8S gel/IFT88 1.tif]

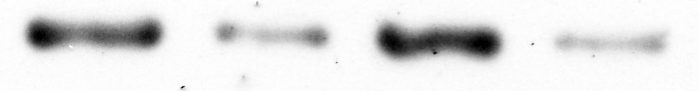

Supplement: Figure 8—figure supplement 1—source data 2. [file elife-73614-fig8-figsupp1-data2.zip › Figure 8S gel/IFT88 2-Gray.tif]

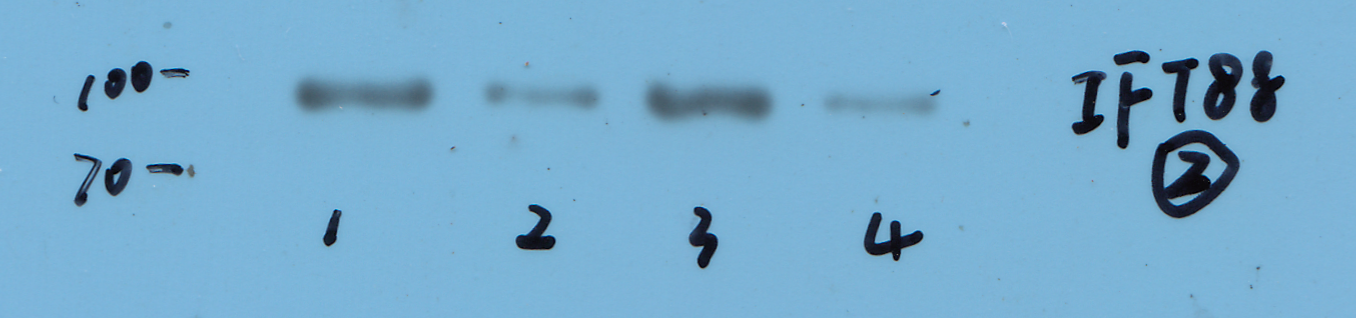

Supplement: Figure 8—figure supplement 1—source data 2. [file elife-73614-fig8-figsupp1-data2.zip › Figure 8S gel/IFT88 2.tif]

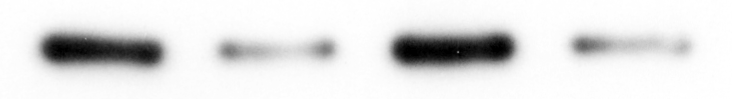

Supplement: Figure 8—figure supplement 1—source data 2. [file elife-73614-fig8-figsupp1-data2.zip › Figure 8S gel/IFT88 3-Gray.tif]

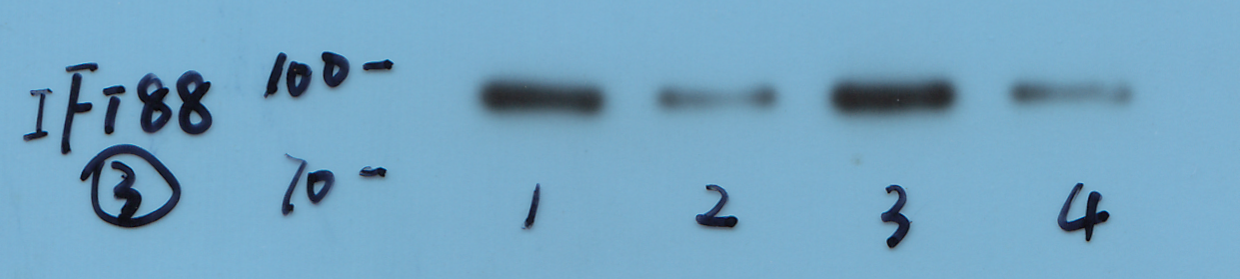

Supplement: Figure 8—figure supplement 1—source data 2. [file elife-73614-fig8-figsupp1-data2.zip › Figure 8S gel/IFT88 3.tif]

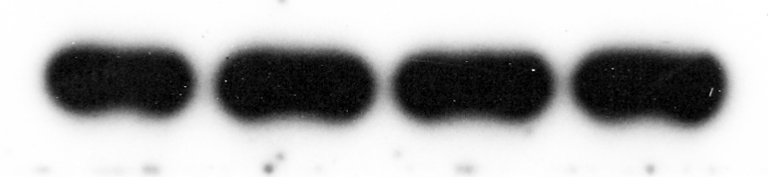

Supplement: Figure 8—figure supplement 1—source data 2. [file elife-73614-fig8-figsupp1-data2.zip › Figure 8S gel/actin 1-Gray.tif]

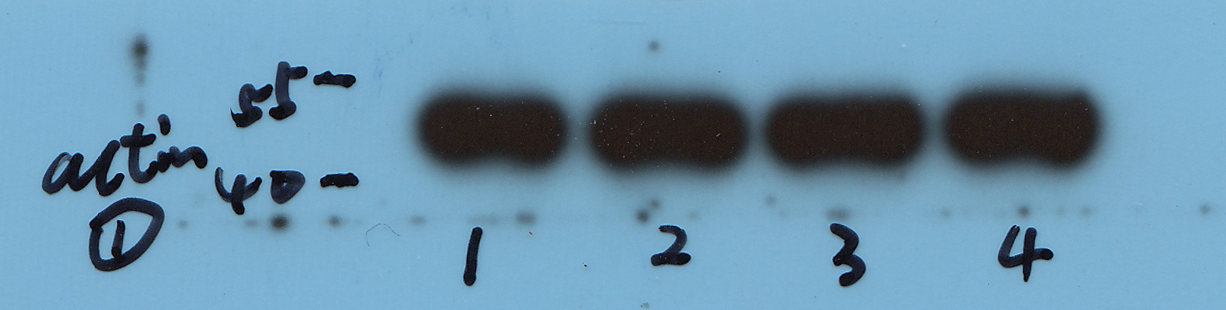

Supplement: Figure 8—figure supplement 1—source data 2. [file elife-73614-fig8-figsupp1-data2.zip › Figure 8S gel/actin 1.tif]

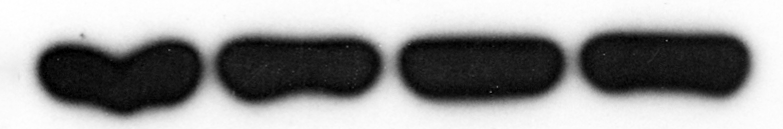

Supplement: Figure 8—figure supplement 1—source data 2. [file elife-73614-fig8-figsupp1-data2.zip › Figure 8S gel/actin 2-Gray.tif]

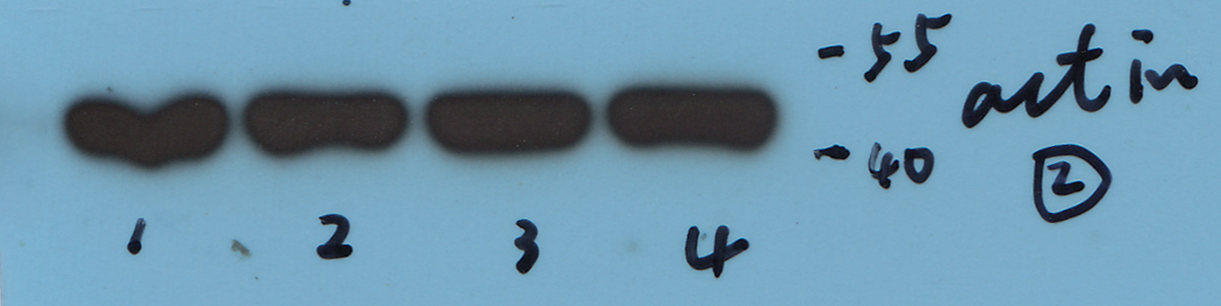

Supplement: Figure 8—figure supplement 1—source data 2. [file elife-73614-fig8-figsupp1-data2.zip › Figure 8S gel/actin 2.tif]

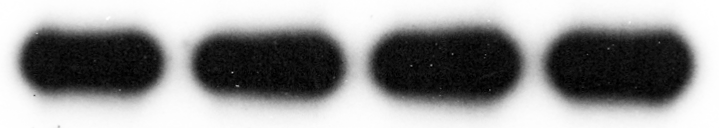

Supplement: Figure 8—figure supplement 1—source data 2. [file elife-73614-fig8-figsupp1-data2.zip › Figure 8S gel/actin 3-Gray.tif]

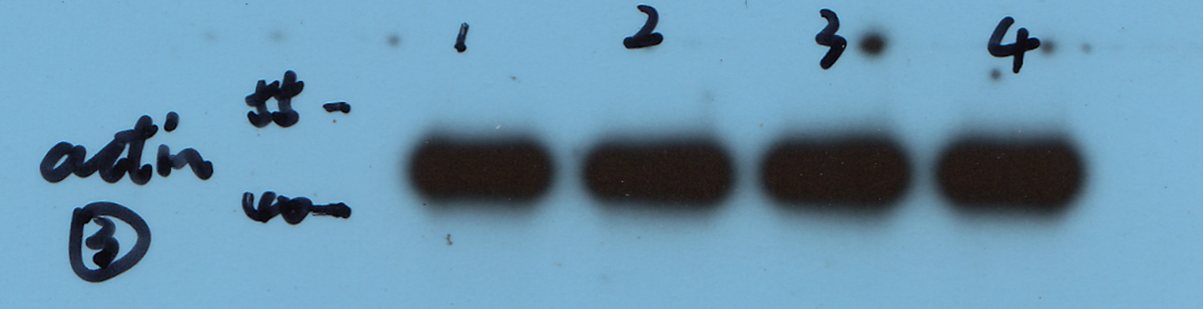

Supplement: Figure 8—figure supplement 1—source data 2. [file elife-73614-fig8-figsupp1-data2.zip › Figure 8S gel/actin 3.tif]
